# Decellularized Extracellular Matrix Scaffolds to Engineer the Dormant Landscape of Microscopic Colorectal Cancer Liver Metastasis

**DOI:** 10.1002/adhm.202501791

**Published:** 2025-10-13

**Authors:** Sabrina N. VandenHeuvel, Lucia L. Nash, Abigail J. Clevenger, Claudia A. Collier, Oscar R. Benavides, Sanjana Roy, Brinlee Goggans, Aelita Salikhova, Anvitha Tharakesh, Svasti Haricharan, Amber N. Stratman, Scott Kopetz, Alex J. Walsh, Shreya A. Raghavan

**Affiliations:** ^1^ Department of Biomedical Engineering Texas A&M University College Station TX 77843 USA; ^2^ Department of Biology San Diego State University San Diego CA 92182 USA; ^3^ Department of Cell Biology and Physiology Washington University School of Medicine St. Louis MO 63110 USA; ^4^ Department of Gastrointestinal Medical Oncology University of Texas MD Anderson Cancer Center Houston TX 77030 USA

**Keywords:** colorectal cancer, dormancy, extracellular matrix, in vitro, liver metastasis

## Abstract

Recurrent liver‐metastatic colorectal cancer contributes to high mortality. Recurrence occurs when dormant, microscopic residual disease survives initial treatment to escape dormancy. In their dormant, microscopic state within the liver, these metastatic lesions are undetectable by clinical diagnostic imaging until they form overt, chemoresistant metastases. Therefore, understanding the molecular mechanisms underlying dormancy in colorectal cancer liver metastases is a significant knowledge gap, motivating the engineering of nuanced in vitro models of disease. The current work presents an engineered model of liver‐metastatic colorectal cancer dormancy. Decellularized extracellular matrix (dECM) scaffolds are used to provide microscopic colorectal cancer cell clusters with a biomimetic, 3D liver‐specific architecture to colonize. Combined with nutrient deprivation and low dose chemotherapy, liver dECM significantly promotes dormancy, which manifests as slowed proliferation, nutrient/chemo‐dependent G1/S and ECM‐driven G2/M cell cycle arrest, diminished tumorigenicity, and robust chemotherapy resistance. The engineered dormancy signature is reversible, mimicking dormancy escape. The dECM‐based model of engineered dormant colorectal cancer liver metastasis is crucial for advancing knowledge of dormancy induction and reversal, to improve therapeutics and patient survival.

## Graphical abstract

Decellularized liver extracellular matrix scaffolds provide a platform to study dormant liver‐metastatic colorectal cancer. They induce reversible dormancy, in combination with nutrient depletion and low dose chemotherapy, through cell cycle arrest and chemotherapy resistance. Mimicking dormancy and recurrence, this model can be used for mechanistic and therapeutic discovery with the goal of improving patient response and survival.

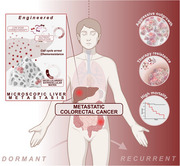

## 1 Introduction

Colorectal cancer is among the most common and deadly cancers in both sexes.^[^ ^1^ ^]^ High mortality is largely ascribed to the metastatic disease that colonizes the liver of 50% of patients. At the time of first diagnosis, synchronous colorectal cancer liver metastases (CRLM) are detected in as high as ≈50% of patients. Due to the aggressive nature of its spread, surgical resection and adjuvant chemotherapy treatment options have suboptimal outcomes in CRLM.^[^ ^2^ ^]^ In the minority of patients where these treatments are effective, 75% still experience recurrence within 2 years with poor survival outcomes.^[^ ^3^, ^4^, ^5^, ^6^, ^7^, ^8^ ^]^

Even during a period of “remission”, many patients unknowingly harbor residual disease, left behind at the site of primary tumor resection or in the form of disseminated colorectal cancer cells that have already seeded into the liver. These microscopic residual cells often exist in a state of dormancy, which allows them to survive by arresting the cell cycle, dampening metabolic rates, and evading immune detection and clearance. Patients remain clinically asymptomatic with these microscopic lesions, and current diagnostic imaging techniques are insufficient to detect them due to their inherently small population size and slowed metabolism.^[^ ^3^, ^9^, ^10^, ^11^, ^12^, ^13^, ^14^ ^]^ Staying below this detection threshold, dormant CRLM can persist for months or even years^[^ ^15^, ^16^, ^17^ ^]^ before restoring growth and forming overt, aggressive, and chemotherapy‐resistant metastases.^[^ ^18^, ^19^ ^]^

Current knowledge of dormant, minimal residual disease CRLM is limited by the clinical inability to detect them via diagnostic imaging prior to their regrowth. The detection limitation leads to insufficient patient data to inform both mechanistic and therapeutic discovery. Major advances in other prevalent and high‐mortality cancer types like breast^[^ ^20^ ^]^ and prostate^[^ ^21^ ^]^ cancer have paved the way toward a better understanding of mechanisms of dormancy induction, maintenance, and reversal. Within hydrogel and organoid models, dormancy manifests as a disrupted cell cycle, which can be measured by expression of cell cycle checkpoint regulators^[^ ^22^, ^23^, ^24^, ^25^, ^26^ ^]^ as well as reduced proliferation^[^ ^22^, ^24^, ^25^, ^27^ ^]^ and metabolism.^[^ ^24^, ^25^, ^27^ ^]^ Literature suggests that the liver microenvironment yields dormant colorectal cancer cells, which are significantly altered compared to their primary tumor counterparts with respect to their production of and interaction with collagen and other extracellular matrix (ECM) proteins.^[^ ^14^ ^]^ The liver niche also contains a unique array of cells, like hepatic stellate cells and Kupffer cells, which contribute to dormant cell survival and escape through soluble signaling factors (ECM proteins, IFN‐γ, CXCL1, etc.) to modulate fibrosis, inflammation, and more.^[^ ^28^, ^29^, ^30^ ^]^ In addition to cancer cell‐stromal cell interactions,^[^ ^31^, ^32^, ^33^ ^]^ interfacing with the ECM is another factor that drives cancer dormancy. ECM organization of metastatic niches, like the liver and brain, for example, can induce dormancy.^[^ ^34^ ^]^ Using in vitro approaches, groups have shown that dense or minimally degradable hydrogel matrices can induce breast cancer dormancy^[^ ^23^, ^35^, ^36^, ^37^, ^38^ ^]^ if cell adhesion molecules like laminin‐211 are available in the metastatic microenvironment.^[^ ^34^ ^]^ Furthermore, pulmonary fibrosis, characterized by type I collagen deposition, caused cytoskeletal reorganization in dormant breast cancer cells, leading to their transition from dormancy to metastatic outgrowth via β1‐integrin signaling.^[^ ^39^ ^]^

Unfortunately, work in colorectal cancer, and specifically its liver‐metastatic form CRLM, has been more limited. Current models utilize methods like nutrient depletion,^[^ ^40^, ^41^, ^42^ ^]^ anti‐cancer drug administration,^[^ ^43^, ^44^, ^45^, ^46^, ^47^, ^48^ ^]^ and genetic manipulation,^[^ ^45^, ^49^, ^50^, ^51^, ^52^, ^53^ ^]^ among others to mimic aspects of dormancy, in vitro. This assortment of dormancy induction methods along with the absence of standardization for metrics to assess dormancy make cross‐study comparisons hard.^[^ ^14^ ^]^ Further, these studies have largely been conducted on monolayer colorectal cancer cell cultures without ECM stimulus. Given the lack of culture models capable of recapitulating the complete CRLM dormancy phenotype (reversible, immune‐evasive, ECM‐influenced^[^ ^54^ ^]^), their biological relevance and translatability is limited. In this study, we aimed to harness the complex 3D architecture unique to the liver using decellularized ECM (dECM) strategies, to create a tissue‐engineered model of CRLM. Using this model, we demonstrate and characterize the induction of CRLM dormancy, with the key variable of the liver ECM intact.

dECM liver scaffolds, specifically those derived from porcine tissue, have been extensively characterized to validate their use in representing healthy human liver. Porosity, heterogeneous architecture, and biochemical composition can be retained through decellularization, allowing the dECM liver to provide a 3D platform uniquely situated to study cellular interactions with biomimetic cues from ECM composition, mechanics, and architecture influencing disease progression.^[^ ^55^, ^56^, ^57^, ^58^ ^]^ Our previous work leveraged dECM liver scaffolds to establish a liver‐metastatic colorectal cancer tissue‐engineered model.^[^ ^57^, ^58^ ^]^In that model, the dECM scaffold was colonized by colorectal cancer cell clusters cultured as spheroids. Successful colonization of the intact 3D liver dECM scaffold was visualized using multiphoton and light sheet microscopy to accurately quantify the number of metastatic cells that invaded into the liver scaffold. The tissue‐engineered CRLM importantly demonstrated a robust resistance to oxaliplatin chemotherapy, compared to spheroid models of colorectal cancer mimicking primary tumor clusters.

Motivated by the knowledge that the ECM is a powerful driver of both cancer metastasis and dormancy, the current work leverages the dECM liver scaffold platform to establish an in vitro model of *dormant* microscopic CRLM. We combined traditional methods of dormancy induction, including nutrient depletion^[^ ^22^, ^41^, ^59^, ^60^ ^]^ and exposure to low dose chemotherapy,^[^ ^44^, ^46^, ^61^ ^]^ with the 3D dECM to demonstrate that the liver ECM enables and promotes dormancy in CRLM. In doing so, our model provides the opportunity to probe molecular mechanisms of CRLM minimal residual disease and dormancy, with the potential to uncover novel diagnostic biomarkers and therapeutic targets to improve patient survival.

## 2 Results

### 2.1 Reduced Serum and Low Dose Chemotherapy Inhibit 3D Spheroid Growth

**Figure 1:**
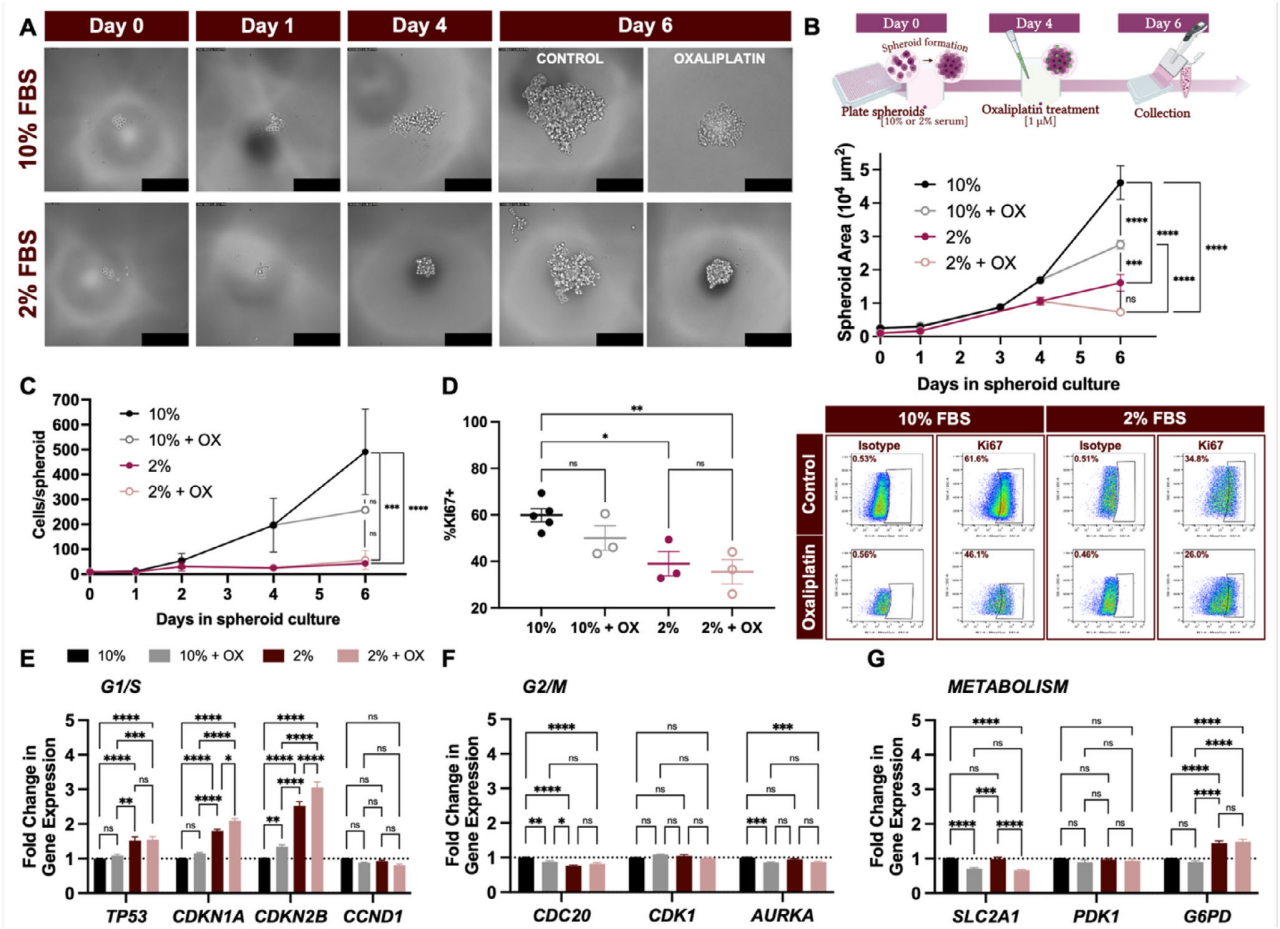
Engineering dormancy in colorectal cancer spheroids. HCT116 colorectal cancer cells were seeded onto the hanging drop array starting at 10 cells/drop in either complete medium (supplemented with 10% FBS) or nutrient‐depleted medium (supplemented with only 2% FBS). A) Phase contrast micrographs show cells upon initial seeding (day 0) settling to the bottom of the drops, followed by clustering and proliferation over 6 days of culture. Nutrient‐deprived spheroids cultured in 2% FBS appeared smaller than 10% FBS spheroids from day 1‐6. Low dose (1 µM) oxaliplatin administered on day 4 also reduced spheroid size in both 10% and 2% FBS spheroids by day 6. Scale bars = 200 µm. B) Top: schematic of the in vitro experimental timeline, showing spheroid seeding and day 4 low dose oxaliplatin administration for dormancy induction. Bottom: visual changes in spheroid size were quantified via projected area from phase contrast micrographs of spheroids. Nutrient depletion alone impeded growth, generating day 6 spheroids that were 60% smaller in 2% FBS than in 10% (^****^ *p*<0.0001, two‐way ANOVA). Low dose oxaliplatin, alone, was also sufficient to reduce spheroid growth (40% smaller with oxaliplatin vs controls, ^****^ *p*<0.0001, two‐way ANOVA). Oxaliplatin also augmented nutrient deprivation‐induced growth arrest (54% size reduction in 2%+OX vs 2%; ns, two‐way ANOVA). C) Orthogonal validation of spheroid size over time included manual cell counts of spheroids dissociated into single cell suspensions. 491.21 ± 171.20 cells were counted per spheroid in the 10% condition reduced to 43.13 ± 10.96 cells per nutrient‐deprived spheroid (^****^ *p*<0.0001, two‐way ANOVA). Although cell count trends followed spheroid size trends, many were not statistically significant (ns, two‐way ANOVA). D) Ki67 protein quantification via flow cytometry also revealed the limited effect of oxaliplatin alone with nonsignificant decreases in the percentage of Ki67^+^ cells (16.5% reduction in 10%+OX vs 10% and 8.9% reduction in 2%+OX versus 2%, ns, two‐way ANOVA). Representative flow cytometry graphs (right) show an isotype control cutoff at ≈0.5% and demonstrate gating strategy used. E) Gene expression of G1/S cell cycle phase regulators revealed serum‐dependent *TP53*, *CDKN1A*, and *CDKN2B* expression with increases in both the 2% condition and 2%+OX over 10% controls (^****^ *p*<0.0001, two‐way ANOVA). Oxaliplatin also contributed to G1/S arrest by upregulating *CDKN2B* (1.34 ± 0.05‐fold in 10%, ^**^ *p*<005; 2.96 ± 0.15‐fold in 2%, ^****^ *p*<0.0001, two‐way ANOVA), and augmenting the effects of nutrient depletion. F) Key regulators of G2/M phase progression were also impacted by dormancy‐inducing stimuli, with oxaliplatin treatment in 10% FBS reducing *AURKA* (^*^ *p*<0.05, two‐way ANOVA). Nutrient deprivation and oxaliplatin both contributed to downshifts in *CDC20* expression (0.87 ± 0.04‐fold change in 10%+OX, ^*^ *p*<0.05; 0.76 ± 0.03‐fold in 2%, ^****^ *p*<0.0001, two‐way ANOVA) but did not compound effects to drive expression further when used in combination (2% vs 2%+OX; ns, two‐way ANOVA). G) Robust metabolic reprogramming was implicated by oxaliplatin‐driven *SLC2A1* reduction (≈30%, ^****^ *p*<0.0001, two‐way ANOVA) and nutrient deprivation‐induced *G6PD* elevation (≈1.5‐fold; ^****^ *p*<0.0001, two‐way ANOVA), both consistent with dormant phenotypes.

HCT116 colorectal cancer cells were seeded into a 384‐well hanging drop array with 10 cells per drop to mimic micrometastatic tumor cell cluster sizes.^[^ ^58^, ^62^ ^]^ Spheroids were cultured in complete medium (supplemented with 10% fetal bovine serum [FBS]) or nutrient depletion medium (supplemented with 2% FBS). The effect of nutrient depletion on spheroid growth was assessed. Representative phase contrast images of spheroids demonstrated the cells settling within the drops by gravity and clustering together to initiate spheroids over the first 24 h (**Figure** **1**A) in both control and nutrient‐depleted conditions. Spheroid size visually increased to indicate cell proliferation. Compact spheroids were established by day 4 in both control and depleted conditions (Figure 1A). The visual progression was supported by two additional readouts: i) quantification of spheroid size via projected area analyses (Figure 1B); and ii) counts of cells per spheroid (Figure 1C) in the complete 10% and nutrient‐depleted, low (2%) serum conditions. Serum‐free (0% FBS) medium failed to sustain spheroids by preventing the cells from initially settling, thereby precluding any clustering or future growth (data not shown).

Spheroids cultured in nutrient‐depleted medium proliferated over time, albeit much more slowly than those in control medium. By day 6, they were significantly smaller (65% smaller; ^****^ *p*<0.0001, two‐way ANOVA, Figure 1B) and contained only a tenth of the cells (43.13 ± 10.96 cells per nutrient‐deprived spheroid) compared to spheroids cultured in complete (10% FBS) medium (491.21 ± 171.20 cells per spheroid; ^****^ *p*<0.0001, two‐way ANOVA, Figure 1C).

At day 4, low dose chemotherapy was used as an additional inducer of dormancy along with nutrient depletion. A subset of spheroids, therefore, in complete or nutrient‐depleted media were additionally treated with oxaliplatin (1 µM, Figure 1B). 48 h of oxaliplatin treatment reduced the size of spheroids cultured in both serum conditions (compare 10% vs 10%+OX and 2% vs 2%+OX). Low dose oxaliplatin augmented the effects of nutrient depletion, manifesting as visible size changes in spheroids and slight decreases in cell counts (Figure 1A–C).

### 2.2 Nutrient Depletion and Low Dose Chemotherapy Slow Cell Cycle Progression of Colorectal Cancer Spheroids

To assay whether the reduction in spheroid size was due to successful induction of dormancy or undesirable cell death, dormancy was evaluated by measuring proliferation and cell cycle checkpoint expression. Depleting nutrients by reducing serum levels from 10% to 2% significantly reduced Ki67 expression (35% reduction; ^*^ *p*<0.05, one‐way ANOVA, Figure 1D), indicating slowed proliferation rather than just cell death. Low dose oxaliplatin in combination with nutrient depletion further slowed proliferation by reducing the population of Ki67^+^ cells (41% reduction; ^**^ *p*<0.01, one‐way ANOVA, compared to control; Figure 1D).

To explore mechanisms behind slowed proliferation (low Ki67 antigen expression), cell cycle checkpoints were first assessed (Figure 1E,F) in control, nutrient‐depleted, and low dose chemotherapy‐exposed spheroids. Nutrient depletion caused increased expression of key regulators of progression through the initial phases (G1/S) of the cell cycle (Figure 1E) whereas low dose oxaliplatin altered genes related to later‐stage G2/M progression (Figure 1F).

Reducing serum from 10% to 2% drastically and significantly increased G1/S regulators *TP53*, *CDKN1A*, and *CDKN2B* (^****^ *p*<0.0001, two‐way ANOVA; Figure 1E). Low dose oxaliplatin without nutrient depletion (10%+OX; grey bars in Figure 1) only resulted in modest changes in G1/S signatures. Combining the two stimuli (2%+OX) produced a strong and significant increase in G1/S checkpoints (*TP53*, *CDKN1A*, and *CDKN2B;* ^****^ *p*<0.0001, two‐way ANOVA, Figure 1E) to indicate cycle arrest. Nutrient depletion and/or low dose oxaliplatin only slightly reduced the G1/S checkpoint, *CCND1*, compared to control spheroids (ns, two‐way ANOVA, Figure 1E).

Regulators of progression through the G2 and M phases were less affected by nutrient depletion and low dose oxaliplatin, where a decrease in the genes of interest would indicate arrest at this phase. Low dose oxaliplatin treatment, but not nutrient depletion alone, lowered *AURKA* expression significantly in both 10%+OX and 2%+OX spheroids (^*^ *p*<0.05, two‐way ANOVA, Figure 1F). On the other hand, nutrient depletion but not oxaliplatin significantly decreased *CDC20* gene expression (20‐30% reduction; ^*^ *p*<0.05, two‐way ANOVA, Figure 1F). *CDK1* was overall unchanged by nutrient depletion or oxaliplatin.

In addition to cell cycle checkpoints, metabolic signatures were also assayed for their dependence on both nutrient depletion and low dose oxaliplatin. Metabolic reprogramming was implicated by significant oxaliplatin‐induced *SLC2A1* reduction (Figure 1G). Though *PDK1* did not appear to be affected by either the serum or oxaliplatin perturbations, *G6PD* increased in the nutrient‐depleted conditions, amplified by low dose oxaliplatin treatment in 2%+OX spheroids (^****^ *p*<0.0001, two‐way ANOVA, Figure 1G).

Withholding nutrients and administering chemotherapy both perturbed cell cycle regulators; with G1/S arrest displaying a large dependence on nutrient access, and oxaliplatin causing some mild G2/M disruption, as well. Through these distinct mechanisms, the two stimuli demonstrate the ability to augment proliferative arrest when used in combination to initiate dormancy in vitro.

### 2.3 Spheroids Demonstrate Functional Dormancy Translating to Delayed Tumorigenicity and Growth Patterns In Vivo

**Figure 2:**
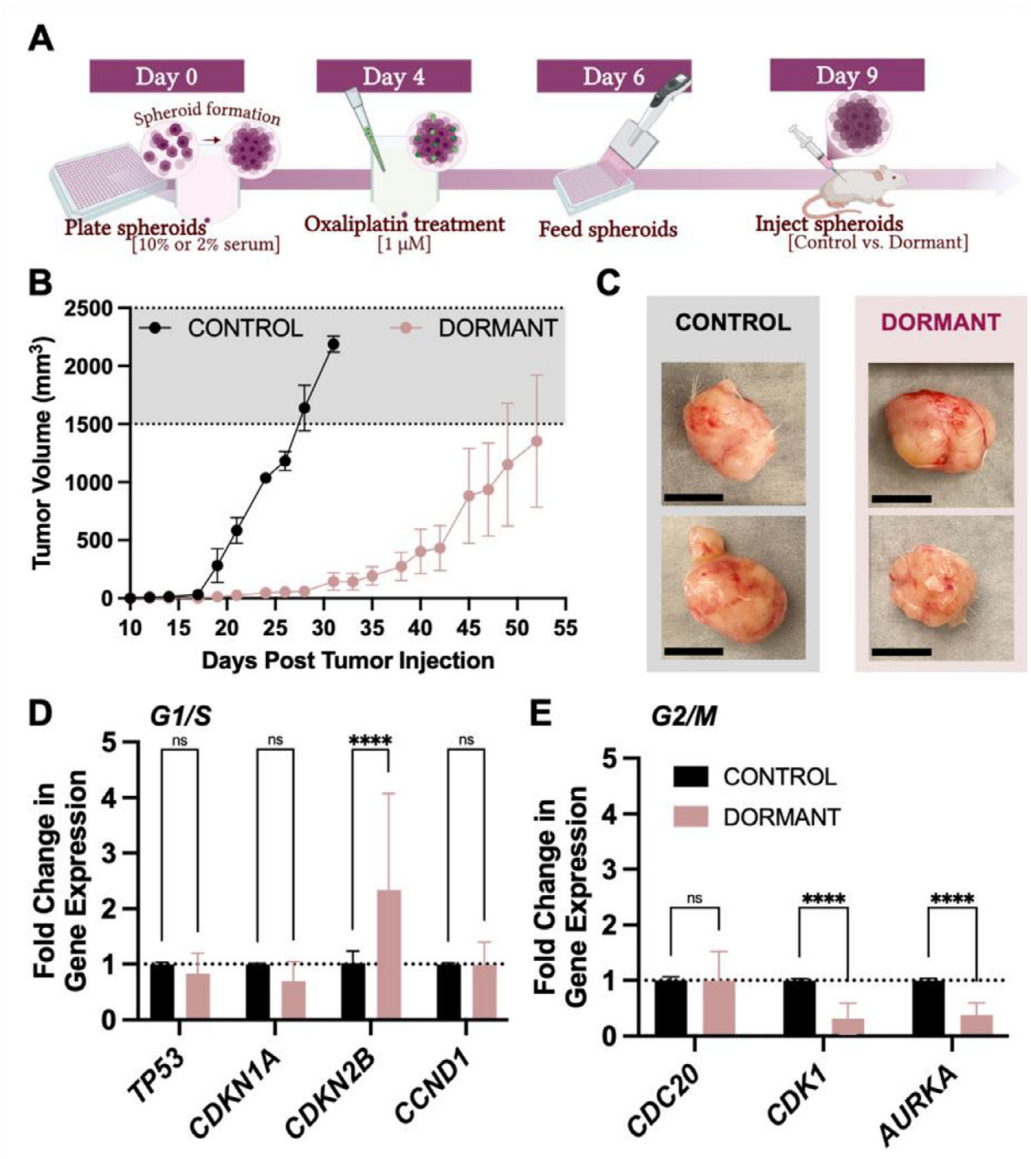
In vivo tumorigenicity of dormant spheroids. A) Schematic depicting generation of control and dormant spheroids, which were cultured for 9 days prior to collection, mechanical dissociation and injection into both flanks of mice at 10 spheroids/tumor. B) Tumor growth was monitored via caliper measurements and calculated volumes, reported as a total tumor burden for each mouse. The shaded region indicates the maximum allowable tumor burden window of 1500–2500 mm^3^. Presentation of control tumors (black trace) was 30% faster than dormant tumors (pink trace), which formed palpable tumors around day 25 compared to day 17 after tumor inoculation in controls. Dormant tumors also grew more slowly, with rates ≈ 13% those of control tumors in the first 10 days post tumor presentation. C) Representative photographs of excised control and dormant tumors show similarly sized, well‐vascularized tumors in both conditions. Tumors were collected on the day that each mouse reached the tumor burden window. The representative tumors shown here were collected on days 31 (top) and 28 (bottom) post tumor cell injection for control, and days 52 (top) and 54 (bottom) post injection for the dormant condition. Scale bars = 1 cm. D) Dormant cells were unable to sustain the *TP53*/*CDKN1A*‐mediated G1/S arrest when grown in mice. However, *CDKN2B* remained elevated over controls (^****^ *p*<0.0001, two‐way ANOVA). G2/M regulators (*CDK1*, *AURKA*) were also significantly altered in dormant tumors compared to control tumors, supporting the cells’ slowed growth in vivo (two‐way ANOVA).

Through in vitro screening, the cooperative effect of nutrient depletion and low dose chemotherapy demonstrated slowed cell cycle progression and proliferation. From these observations, the 2%+OX condition was considered “dormant” for future studies and compared to the control (10% serum) condition. A functional phenotype of the dormant spheroids was assessed by their capacity to establish xenograft tumors in mice over 60 days. 10 spheroids (control or dormant) were used to initiate flank tumors in mice (**Figure** **2**A), with subsequent growth follow‐up for 60 days. Cell counts per spheroid were estimated experimentally in data shown in Figure 1C. Control spheroids prepared in 10% medium established tumors faster (averaging 17.5 days between injection and tumor presentation). Dormant spheroids were significantly slower, with average initiation times closer to 24.8 days (^*^ *p*<0.05, unpaired t test, Figure 2B). Dormant tumors also grew at a much slower rate (7.66 ± 2.40 mm^3^/day) in the first 10 days after tumor presentation compared to controls (59.61 ± 13.81 mm^3^/day; ^**^ *p*<0.01, unpaired t test, Figure 2B).

The slower growth was supported by the gene profiles of xenografted tumors established from dormant spheroids compared to control spheroids (Figure 2C). Dormant xenograft tumors did not demonstrate significant differences in many of the G1/S cell cycle checkpoints except *CDKN2B* (Figure 2D). Interestingly, dormant xenograft tumors had a significant shift toward G2/M cell cycle arrest (Figure 2E) with reductions in *CDK1* and *AURKA*. Overall, these data supported functional dormant behavior resulting from nutrient‐ and drug‐dependent dormancy established in hanging drop spheroids.

### 2.4 Decellularized Liver ECM Scaffolds Sustain and Amplify Cell Cycle Arrest and Colorectal Cancer Dormancy

**Figure 3:**
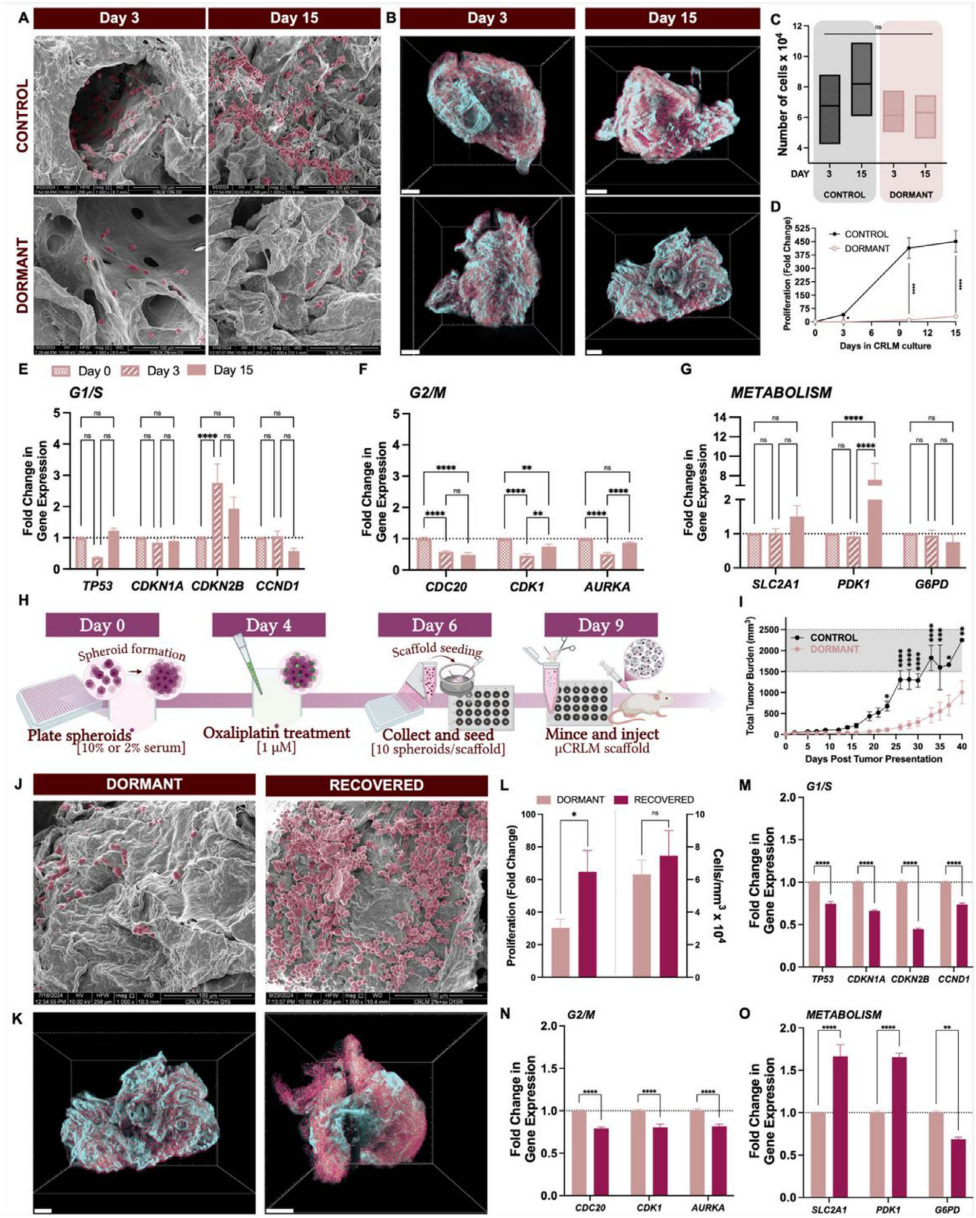
Sustained dormancy in microscopic colorectal cancer liver metastasis (µCRLM) engineered in dECM scaffolds. Control and dormant spheroids were collected and seeded onto decellularized liver ECM scaffolds to establish in vitro µCRLM. A) Scanning electron micrographs show dECM scaffolds were successfully colonized by both control and dormant cells, though fewer cells are visible in dormant samples (cells psuedocolored maroon). With time, increased cellular occupancy was observed in control µCRLM, but dormant population size remained relatively constant through day 15. Scale bars = 100 µm. B) Whole‐mount light sheet fluorescent microscopy (LSFM) enabled visualization of the entire scaffold volume and architecture via autofluorescence (pseudocolored cyan) and fluorescent detection of cells (pseudocolored maroon). Scaffold occupation appeared to increase from day 3 to day 15 in control but not dormant µCRLM. Scale bars = 500 µm. C) Cell counts from segmented LSFM images (normalized to scaffold volume) were 1.2 times higher at day 15 compared to day 3 in control samples (ns, unpaired t test). Dormant cell populations were comparatively unchanged over the same timespan (ns, unpaired *t* test). D) Proliferation was orthogonally assessed via viability measurements. By day 3 in µCRLM culture, dormant cells showed reduced proliferation, at less than 10% the rate of control cells through day 15 (^****^ *p*<0.0001, two‐way ANOVA). E) G1/S phase regulator gene expression was mostly unaffected by introduction of dECM except by *CDKN2B*, which increased 2.8‐fold at day 3 (^****^ *p*<0.0001) and sustained twofold elevation through day 15 (ns, two‐way ANOVA) over day 0 (dormant spheroids). F) G2/M arrest was implicated in the gene signature of µCRLM samples, most prominent at day 3 upon initial colonization of the scaffolds (^****^ *p*<0.0001, two‐way ANOVA). *CDC20* expression remained low through day 15 (^*^ *p*<0.05, two‐way ANOVA), but *CDK1* and *AURKA* experienced recovery of this signature over time. G) Metabolic genes did not immediately indicate slowed metabolism but displayed a strong sign of metabolic dysregulation or reprogramming through a 7.6‐fold increase in *PDK1* expression (^****^ *p*<0.0001, two‐way ANOVA). H) A timeline schematic shows that µCRLM scaffolds seeded with control or dormant spheroids were cultured for 3 days prior to collection for in vivo experiments. Colonized scaffolds were minced and injected to form flank tumors in mice. I) Control tumors grew significantly faster than dormant tumors, evidenced by tumor burdens ≈ 8 times that of mice bearing dormant tumors (^****^ *p*<0.0001, unpaired t test). J) Scanning electron micrographs present the result of removing dormancy‐inducing medium to allow for recovery of dormant µCRLM scaffolds. The few cells visible in the dormant scaffold appear in stark contrast to the populated scaffold, indicating immediate return to proliferation upon nutrient restoration and oxaliplatin withdrawal. Scale bars = 100 µm. K) LSFM volumes further demonstrated this trend of increasing scaffold occupation following recovery (scaffold, cyan; cells, maroon). Scale bars = 500 µm. L) Proliferation was quantified by viability measurements which significantly increased after recovery compared to dormancy (^*^ *p*<0.05, unpaired t test), and by cells identified in LSFM image data (ns, unpaired t test) showing the same increasing trend upon recovery, though to a lesser degree. M) Nutrient restoration and oxaliplatin withdrawal reversed G1/S arrest induced by dormancy medium (*TP53*, *CDKN1A*, *CDKN2B*, *CCND1*; ^****^ *p*<0.0001, two‐way ANOVA). N) The G2/M arrest signature originally induced by ECM introduction interestingly did not demonstrate reversal following recovery. Rather, this ECM‐regulated arrest was maintained and the effects intensified in the absence of dormancy‐inducing medium (*CDC20*, *CDK1*, *AURKA*; ^****^ *p*<0.0001, two‐way ANOVA). O) Metabolic trends also supported a return to proliferative competency via *SLC2A1* and *PDK1* increasing 1.7‐fold (^****^ *p*<0.0001) and a 0.7‐fold decrease in *G6PD* expression (^**^ *p*<0.005, two‐way ANOVA).

Next, the ability of the liver extracellular matrix (ECM) microenvironment to sustain a dormant phenotype was tested. Spheroids cultured in either control medium (10% serum) or in dormancy‐inducing medium (2% serum + oxaliplatin) were collected and seeded onto each decellularized ECM (dECM) liver scaffold to establish microscopic colorectal cancer liver metastasis (µCRLM). Representative scanning electron micrographs demonstrated colorectal cancer cell adhesion to the scaffold by day 3 of µCRLM culture in both control and dormant conditions (**Figure** **3**A; cells pseudocolored maroon). Due to smaller spheroid size, the initial seeding density of dormant cells into the dECM scaffold was lower than that of control (≈569 cells/scaffold compared to 4912 in controls; Figure 1C). As such, control µCRLM samples harbored more cells than dormant samples, specifically at the early timepoint. By day 15, many more cells were visible in control samples, indicating robust proliferation and scaffold colonization over time. In contrast, micrographs of dormant µCRLM revealed a much smaller cell population, even at the day 15 timepoint (qualitative visual observation, Figure 3A).

To orthogonally assess scaffold colonization, we first employed an imaging method that allowed the visualization of cells within the entire scaffold volume through light sheet fluorescence microscopy (LSFM). Representative images demonstrating colonization are shown in Figure 3B, where the entire dECM scaffold was visualized in cyan, with automatically segmented cells appearing in maroon. To identify changes between control and dormant µCRLM cultures, cell numbers were quantified from LSFM images between day 3 and day 15 of culture on dECM liver scaffolds. Interestingly, the rate of change in the number of colonized cells was much higher in control cultures, compared to dormant µCRLM cultures (compare 21% increase in control versus 3% in dormant µCRLM; Figure 3C), indicative of slowed proliferation.

The second orthogonal method used the CellTiter Glo ATP reporter assay, demonstrating significantly inhibited proliferation rate between control and dormant µCRLM cultures within dECM liver scaffolds (compare 451‐fold increase in control to 30‐fold increase in dormant cultures; ^****^ *p*<0.0001, two‐way ANOVA, Figure 3D). To exclude the possibility that this difference resulted from lower initial seeding density, parallel experiments were performed with equal starting cell numbers from control and dormant cultures. Growth monitoring over 15 days again showed that dormant culture conditions significantly reduced proliferation compared to controls (Figure S1, Supporting Information). Together, our data demonstrated the ability of the dECM scaffold to support dormant colonization, generating in vitro microscopic colorectal cancer liver metastases.

### 2.5 Liver ECM Contributes to Functional Colorectal Cancer Dormancy

The role of the dECM in not only permitting but contributing to this dormant behavior was investigated next. Analysis of key cell cycle (Figure 3E,F) and metabolic process (Figure 3G) genes revealed limited change in G1/S (Figure 3E), but a robust response by G2/M regulators to matrix introduction and colonization (Figure 3F). Importantly, these signatures shifted with increased culture time in the dECM scaffolds.

The presence of liver dECM significantly disrupted G2/M phase regulators (Figure 3F) toward cycle arrest. Upon initial seeding and 3 days of µCRLM culture, expression of *CDC20, CDK1*, and *AURKA* decreased ≈50% from day 0 (^****^ *p*<0.0001, two‐way ANOVA, Figure 3F). While *AURKA* gene expression recovered by day 15 of µCRLM culture, *CDK1* and *CDC20* failed to recover original expression and remained suppressed across 15 days of µCRLM culture (^****^ *p*<0.0001, two‐way ANOVA, Figure 3F).

Metabolic reprogramming was also affected by the liver dECM environment, not initially within 3 days of culture, but over time. No change in metabolic activity occurred over the first 3 days (ns, two‐way ANOVA, Figure 3G). By day 15, gene expression shifted, only slightly for *SLC2A1* and *G6PD* but significantly by 7.60 ± 1.68‐fold for *PDK1* (^****^ *p*<0.0001, two‐way ANOVA, Figure 3G), showing a stark change in metabolic activity as the samples colonized and invaded the scaffolds with time. Analysis of cell cycle and metabolic gene profiles indicated the ability of the liver dECM scaffold to both i) sustain spheroid‐induced dormancy by maintaining or intensifying G1/S phase gene levels and ii) contribute to the dormancy signature by imposing additional cell cycle disruptions demonstrated by significant G2/M gene disruption and metabolic reprogramming. Validation experiments further confirmed that the dECM alone was sufficient to promote dormancy via G2/M disruption in the absence of dormancy‐inducing medium (Figure S2, Supporting Information).

Next, the functional realization of this dormant signature was probed by inoculating mice with µCRLM (microscopic colorectal cancer liver metastases engineered within liver dECM scaffolds) to establish flank tumors. Figure 3H shows the timeline of generating control or dormant spheroids and seeding them onto liver dECM scaffolds. After 3 days of scaffold colonization, 1 control or dormant µCRLM sample was minced and injected to form a tumor. The total tumor burden per mouse over 40 days following tumor presentation is demonstrated in Figure 3I. Growth curves were normalized to days post tumor presentation within each group rather than injection day to focus on growth rate as opposed to differences in presentation time, which could be skewed by differences in injected cell numbers. These normalized data demonstrate that tumors from control µCRLM grew significantly faster and larger than tumors initiated from dormant µCRLM (compare 677.96 ± 113.45 mm^3^ in control versus 111.12 ± 41.39 mm^3^ in dormant on day 23, ^*^ *p*<0.05, and 2253.46 ± 0.00 mm^3^ control tumors versus 1004.62 ± 272.88 mm^3^ dormant tumors on day 40, ^**^ *p*<0.005, two‐way ANOVA, Figure 3I). Control mice measured at greater than 100 mm^3^ by day 9, as opposed to dormant mice, which reached that threshold on day 23 prior to an apparent dormancy escape and tumor outgrowth. This functional assessment supported the dormancy‐indicating metrics used to evaluate the dormant phenotype of colorectal cancer cells within in vitro µCRLM scaffolds. It also reinforced the ability of the in vitro model to be used to mimic and study dormant behavior.

### 2.6 Dormant µCRLM can Escape Dormancy and Exhibit Growth Recovery

Dormant cell re‐entry into the cell cycle mimics disease recurrence in colorectal cancer. Since the dECM liver scaffolds induced and supported dormancy, we next evaluated if they would support cell cycle re‐entry. To test this competency, nutrient access was restored and low dose oxaliplatin was withdrawn at day 10 of µCRLM, to allow for 5 days of recovery. Immediate cell proliferation was evident from electron micrographs and LSFM images, where cellular occupancy markedly increased compared to dormant µCRLM cultures (Figure 3J,K). Visual data was supported by significant increases in measured proliferation rates (^****^ *p*<0.0001, two‐way ANOVA, Figure 3L), and slight increases in cells occupying the scaffold compared to dormant time‐matched µCRLM cultures (Figure 3C).

Interestingly, restoring nutrient access and withdrawing chemotherapy robustly reversed the G1/S arrest gene signature. Significant reductions in *TP53* (25%), *CDKN1A* (34%), and *CDKN2B* (55%) indicated release from the G1/S growth restriction (^****^ *p*<0.0001, two‐way ANOVA, Figure 3M). Importantly, despite this restoration of nutrient access and oxaliplatin withdrawal, G2/M signatures did not reverse, and in fact significantly amplified (^****^ *p*<0.0001, two‐way ANOVA, Figure 3N).

Nutrient access restoration and oxaliplatin withdrawal similarly resulted in significant reversal of metabolic trends compared to time‐matched dormant µCRLM cultures (^****^ *p*<0.0001, two‐way ANOVA, Figure 3O).

Collectively, our data continues to underscore that the presence of dECM liver scaffolds sustained dormancy due to matrix effects on G2/M, rather than cell cycle checkpoint inhibition of G1/S. Despite recovery of metabolism upon restoration of nutrient access and oxaliplatin withdrawal, dECM presence continued to drive G2/M arrest in recovered µCRLM cultures. These data also support a reversible dormancy signature, with the ability to controllably induce re‐entry into the cell cycle following a period of dormancy in the µCRLM model.

### 2.7 Unbiased Transcriptomics Emphasize Key Aspects of the Dormancy Signature Imposed by Nutrient Depletion, Chemotherapy, and dECM Scaffolds

**Figure 4:**
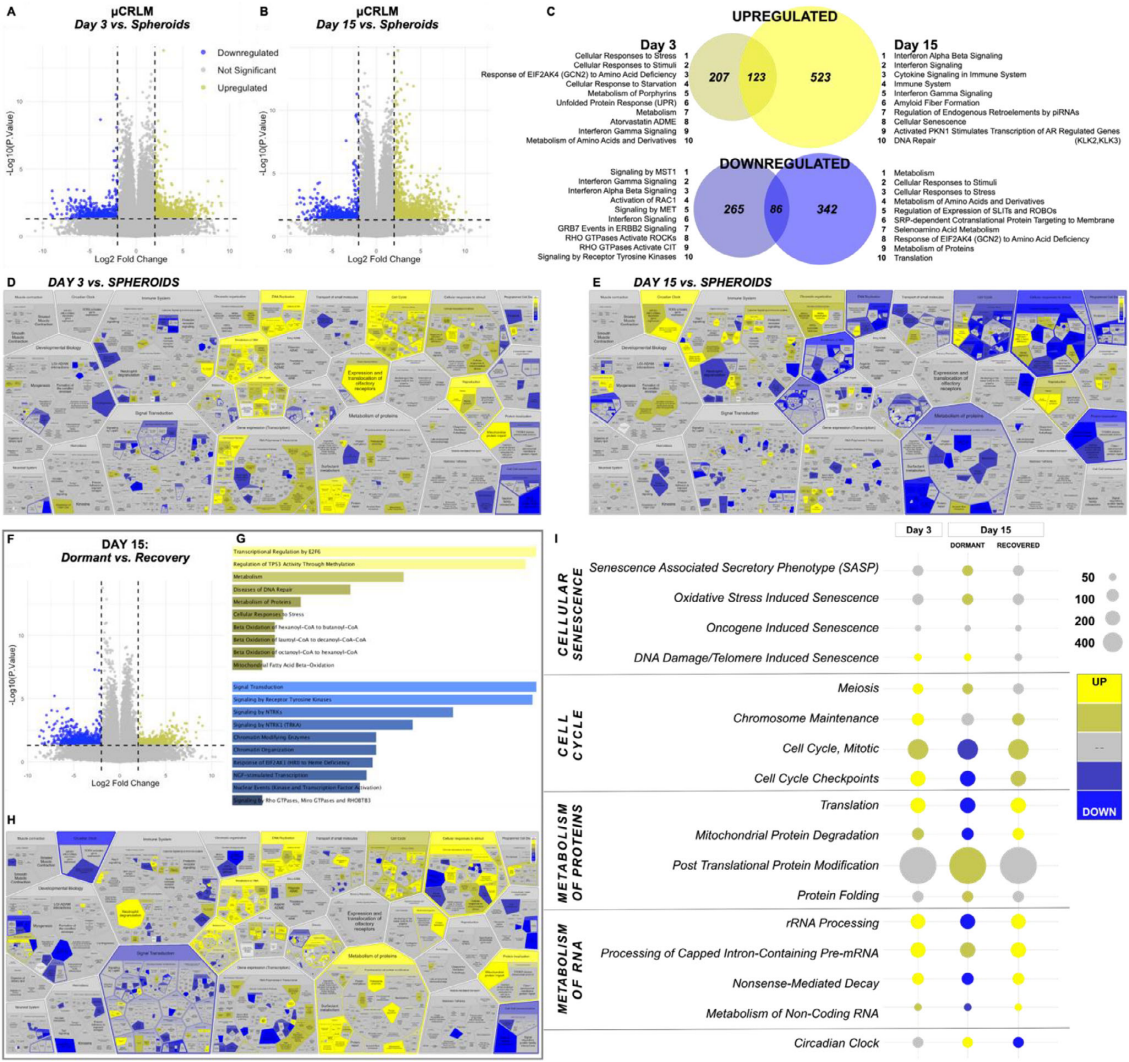
Unbiased RNA sequencing also demonstrates increasing dormancy in engineered µCRLM within dECM scaffolds. A) Volcano plots depict large‐scale differences in global gene expression when day 3 or B) day 15 µCRLM was compared to dormant spheroids. Blue dots signify genes downregulated (log2fold change ≥ 2) with significance (p‐value < 0.05) in the µCRLM conditions, and yellow dots represent significantly upregulated genes compared to spheroids. C) Differentially expressed genes identified at each timepoint were compared and visualized in a Venn diagram to demonstrate the number of unique genes upregulated (top) or downregulated (bottom) in each condition. These unique genes were used to identify the top 10 enriched Reactome processes unique to each comparison (µCRLM day 3 vs spheroids or µCRLM day 15 vs spheroids). D) Voronoi diagrams represent the Reactome pathway analysis for the comparison of day 3 µCRLM or E) day 15 µCRLM to spheroids to show significantly upregulated (yellow) and downregulated (blue) pathways. F) Global gene expression of recovered µCRLM at day 15 was compared to that of dormant µCRLM at the same timepoint, and differentially expressed genes were displayed with a volcano plot. G) The top 10 upregulated (top, yellow) and downregulated (bottom, blue) Reactome processes were also identified for the recovered conditions compared to dormancy. H) The Voronoi diagram represents general changes resulting from dormancy escape. Many of the processes significantly altered when dormant day 15 µCRLM was compared to spheroids (Figure 4E) appear to be reversed by the recovery period (i.e., blue areas changing to yellow with recovery). I) Pathways of interest related to cellular dormancy, survival, and metabolism were identified in the Reactome analysis and examined in closer detail with a dot plot, which indicates upregulation and downregulation as well as significance with color (yellow: upregulation, grey: no change, blue: downregulation). Within each of the biological processes, the number of genes altered within each pathway is indicated by the size of the corresponding dot in the plot. Day 3 µCRLM showed ECM introduction induced stress response and increased metabolism and overall cellular activity. Day 15 µCRLM, on the other hand, showed signs of senescence, reduced metabolism, and dysregulated circadian clock to support dormancy. Many of these trends can be seen reversed when µCRLM was allowed to recover from dormancy.

Gene expression of dormant µCRLM scaffolds (day 3, day 15) was compared to dormant spheroids to query the effects of liver ECM in the µCRLM model. The analysis yielded a dormancy signature which evolved throughout in vitro µCRLM experimental timelines between 3 and 15 days. This signature included 681 uniquely and significantly differentially expressed genes at day 3 (**Figure** **4**A) and 1,074 genes at day 15 (Figure 4B) compared to spheroids. The difference in the number of unique genes clearly indicated that the presence of the dECM scaffold modified the biology of spheroids, adapting into µCRLM culture.

An unbiased pathway analysis was performed on the unique genes to identify the top 10 pathways that were up‐ or down‐regulated uniquely at day 3 µCRLM and day 15 µCRLM (Figure 4C). Notably, cellular responses to stress, unfolded protein response and metabolism were all upregulated at day 3, when spheroids are in the initial stages of colonizing the dECM scaffold. By day 15 of µCRLM culture, this signature had shifted. Upregulation of cellular senescence and interferon and cytokine signaling was observed, accompanied by a decrease in metabolism (Figure 4C).

Based on the distinct gene expression changes at the global level, we applied a Reactome Camera analysis to identify specific processes altered by the unique differential gene sets. This analysis revealed large‐scale transcriptomic alteration in response to the dECM scaffold environment shown by Voronoi diagrams highlighting many upregulated pathways at day 3 (yellow, Figure 4D) and subsequent significant downregulation at day 15 (blue, Figure 4E). In fact, the reversible nature of dormancy was also evident in the unbiased transcriptomic analysis of the day 15 µCRLM in recovery, where nutrient access was fully restored and oxaliplatin was withdrawn. Volcano plots indicate 309 genes up and 600 genes down in recovered day 15 µCRLM, compared to dormant day 15 µCRLM (Figure 4F). The Voronoi diagrams of recovered day 15 µCRLM presented with more apparent upregulation (yellow, Figure 4H) in metabolism and several other biological pathways that were lower in dormant day 15 µCRLM.

Due to the significant differences in transcriptomic signatures resulting from the presence of liver dECM in dormant µCRLM, pathways of interest between day 3, dormant day 15 and recovered day 15 µCRLM were evaluated in closer detail. These selected pathways included cellular senescence, cell cycle, metabolism of proteins, metabolism of RNA, and circadian clock. Within each of these selected pathways, differentially expressed gene counts are displayed by the dot plot in Figure 4I for direct comparison across the timepoints. At day 3, signs of cellular senescence were overall unchanged compared to dormant spheroids, with some increase in the downstream effect of low dose oxaliplatin‐induced DNA damage and resulting senescence. By day 15, µCRLM took on a stronger secretory senescent signature, upregulating the senescence‐associated secretory phenotype (SASP) and oxidative stress pathways. Signatures associated with cellular senescence disappeared in recovered day 15 µCRLM compared to dormant µCRLM, indicating the reversible nature of cellular senescence induced by long‐term culture in dECM scaffolds.

Seeding dormant colorectal cancer cells onto dECM liver scaffolds caused initial stimulation of cell cycle checkpoints as well as mitotic and meiotic processes (day 3, Figure 4I). Following longer incubation in the scaffold, the cells settled into a dormant signature with key shifts in cell cycle checkpoints and mitotic cell cycle regulators to indicate their arrest (day 15, Figure 4I), which was reverted upon recovery at day 15 when nutrient access was restored and oxaliplatin was withdrawn.

µCRLM metabolism was significantly increased upon the initial introduction of the new ECM environment (day 3) during the colonization phase. This was evident via increases in protein translation and degradation, with no changes in post translational protein modifications. In contrast, however, continued and prolonged dECM scaffold culture (day 15) downregulated both protein translation and degradation with a corresponding increase in post translational protein modification, indicative of senescent and metabolically dysregulated adaptation. These trends were again reversed upon restoration of nutrient access and oxaliplatin withdrawal, during recovery. Changes in protein metabolism were paralleled by RNA metabolism, with upregulation at day 3, downshifts at day 15 and subsequent reversal with recovery.

Importantly, the Reactome pathways of interest (Figure 4I) revealed expression changes for many of the genes from the targeted qPCR gene expression analysis shown in Figures 1, 2, 3 (Figure S3, Supporting Information). Unbiased transcriptomic analyses clearly validate liver dECM scaffolds as an influential modulator of colorectal cancer cell proliferation, senescence, and metabolism with global alterations in many signaling pathways and biological processes at both the day 3 and day 15 timepoints compared to dormant spheroids.

### 2.8 Dormant µCRLM Signature Displays Functional Significance via Chemoresistance

A hallmark of dormant CRLM is enhanced chemotherapy resistance compared to its control, non‐dormant counterpart. As such, we tested the response of our dormant model to oxaliplatin chemotherapy to evaluate this hallmark.

**Figure 5:**
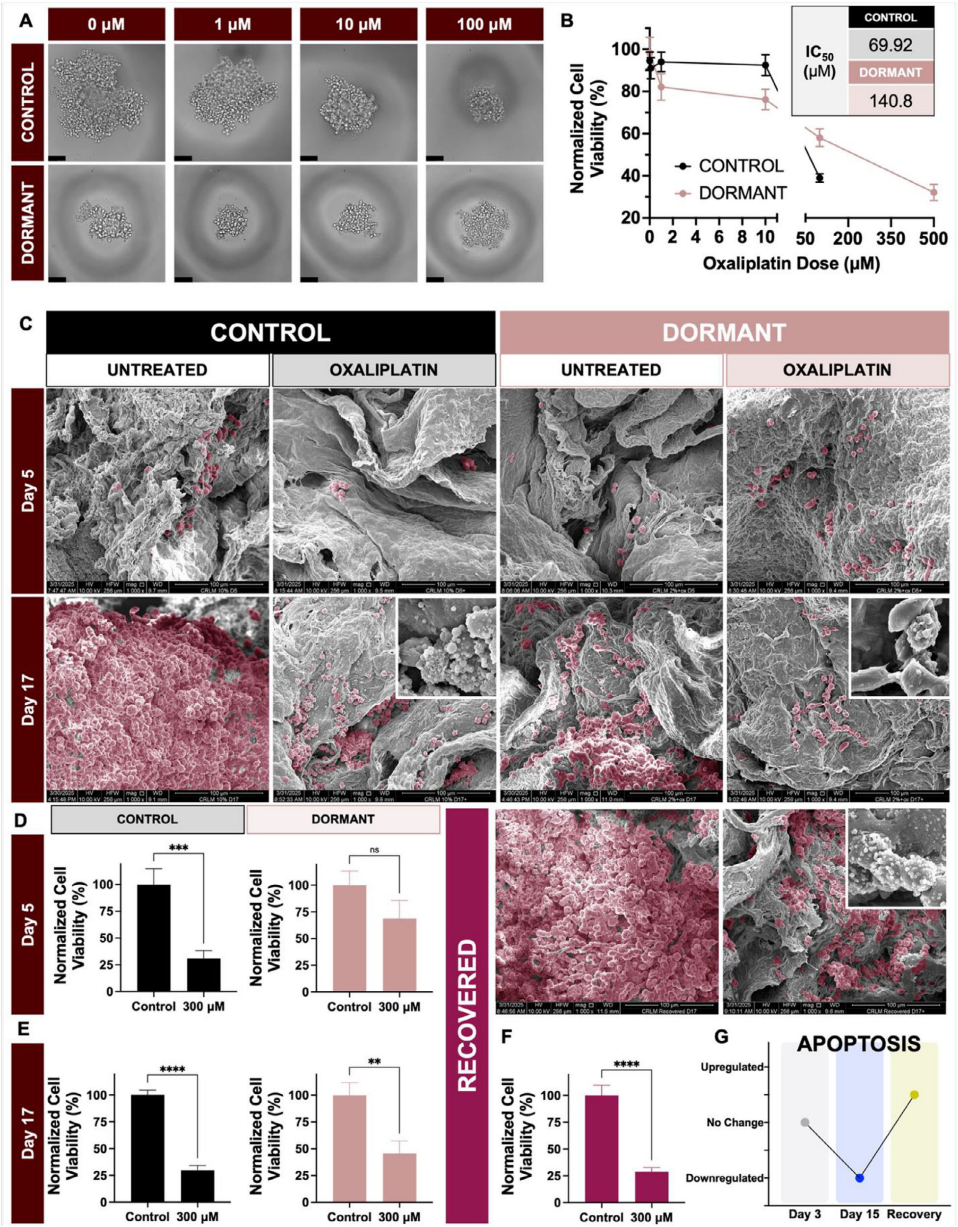
Functional survival and chemoresistance of dormant µCRLM. A) Hanging drop spheroids cultured in control (10% FBS) or dormant (2% FBS + day 4 oxaliplatin) were challenged with oxaliplatin chemotherapy at a range of doses (0‐500 µM) on day 6. Phase contrast images on day 8 (after 48‐h incubation with the drug) display the expected size difference of dormant spheroids smaller than controls with 0 µM oxaliplatin addition. With increasing dose, control spheroids (top) get smaller and are surrounded by increased cellular debris. Cell morphology is also affected, particularly notable in images of cells treated with 100 µM oxaliplatin, which show less circular cells and less defined spheroid boundaries. In contrast, oxaliplatin treatment did not appear to shrink dormant spheroid, despite their initial small size. Less debris and cell morphology disruptions are visible at 100 µM compared to controls. B) Normalized viability measurements demonstrate the percentage of viable cells dropping more quickly in controls compared to dormant spheroids to produce an IC_50_ value of 70 µM in control spheroids compared to 141 µM in dormant spheroids. C) Control and dormant spheroids were used to colonize µCRLM scaffolds. 300 µM oxaliplatin was then administered to the scaffolds on day 3 or day 15, and scaffolds were incubated for 2 more days. This concentration was chosen to intentionally increase from the IC_50_ value calculated in spheroids, accounting for diffusional limitations within the scaffold model. Scanning electron micrographs represent the occupation of the scaffolds at days 5 or 17, both after 2 days of incubation with the drug. Following oxaliplatin treatment at both timepoints, control cell populations decreased drastically. Cell death is visible in the zoomed inset at day 17 by increased presence of texture in the images representing apoptotic bodies and cellular debris. Dormant populations were comparatively less affected by oxaliplatin, showing no reduction at day 5 and some at day 17. The zoomed inset at day 17 shows a comparative lack of apoptotic bodies and debris in dormant µCRLM to support this observed resistance. After recovery, dormant µCRLM increased proliferation, evidenced by increased occupation of scaffolds in untreated, day 17 images. They also appeared to regain sensitivity to chemotherapy with drastic decreases in cellular occupancy of scaffolds and increased apoptosis visible in the zoomed inset. D) Visual changes were confirmed by viability measurements of cells within the µCRLM scaffolds. Control cell viability was significantly reduced by oxaliplatin (^***^ *p*<0.001, unpaired t test) while dormant populations were relatively unaffected and showed only slight reduction in viability after treatment. E) At day 15, control cells similarly showed a strong death response to oxaliplatin (^****^ *p*<0.0001) whereas the response was dampened in dormant µCRLM (^**^ *p*<0.005, unpaired t test). F) Oxaliplatin was more efficient at killing recovered CRLM, demonstrating the reversible chemoresistant dormancy phenotype induced by the dormancy medium. G) Unbiased transcriptomic analysis of µCRLM scaffolds revealed significant changes in the apoptotic process when µCRLM was compared to spheroids. Specifically, with prolonged culture in dECM scaffolds, day 15 µCRLM significantly downregulated biological processes related to apoptosis, which were reinstated after dormancy escape, corresponding to chemotherapy resistance mechanisms implicated by proliferation results.

Colorectal cancer spheroids were cultured in control or dormancy‐inducing medium (2%+OX). Cell viability was determined following oxaliplatin treatment (0–500 µM) in both control and dormant spheroids. Interestingly, phase contrast micrographs demonstrated a significant reduction in spheroid size when control spheroids were treated with oxaliplatin, compared to dormant spheroids treated with oxaliplatin (qualitative visual observation, **Figure** **5**A). Chemo‐induced death was further indicated by increased cellular debris surrounding the control spheroids and individual cells appearing less circular. Dormant spheroids, in contrast, retained relative integrity under oxaliplatin challenge. Quantification of cell viability across control and dormant spheroids demonstrated chemoresistance (Figure 5B). An increased oxaliplatin IC_50_ was observed in dormant spheroids compared to control spheroids (compare 140.8 µM in dormant vs 69.9 µM in control).

Chemoresistance was next evaluated in control or dormant µCRLM cultures that were challenged with 300 µM oxaliplatin for 48 h on either day 3 or day 15. Scanning electron microscopy (SEM) images verified the expected proliferation of control cells from day 5 to day 17 in stark contrast to dormant µCRLM, which grew more slowly across this timeline (Figure 5C). Oxaliplatin treatment resulted in significant reduction in cellular occupancy of control µCRLM scaffolds, indicating cell death in response to drug treatment. In comparison, fewer cells were “lost” upon oxaliplatin treatment in dormant µCRLM cultures. Interestingly, at day 17, control non‐dormant µCRLM cultures displayed more signs of apoptosis upon oxaliplatin treatment compared to dormant µCRLM, evident from the high magnification image insets on the SEM micrographs (Figure 5C, high mag zoomed insets).

Orthogonal validation of viability via CellTiter Glo assays continued to demonstrate similar trends with oxaliplatin treatment between control non‐dormant and dormant µCRLM cultures (Figure 5D,E). Oxaliplatin treatment reduced viability significantly by ≈70% at both timepoints in control µCRLM cultures (^***^ *p*<0.001, ^****^ *p*<0.0001, unpaired t test, Figure 5D,E). In contrast, oxaliplatin treatment only slightly reduced viability in dormant µCRLM cultures at day 5 (≈32% reduction). Due to gradual outgrowth, by day 17, dormant µCRLM became slightly more responsive to chemotherapy, experiencing a more significant 55% loss of viability (^**^ *p*<0.005, unpaired t test, Figure 4E). Despite this responsiveness, dormant µCRLM was still more resistant to oxaliplatin treatment compared to time‐matched non‐dormant control cultures. Upon withdrawal of dormancy‐inducing medium at day 10, recovered µCRLM treated with oxaliplatin on day 15 responded more strongly to the drug, which killed 71% of the recovered cells (^****^ *p*<0.0001, unpaired t test, Figure 5F). These trends indicated that dormancy reversal in µCRLM cultures also enhanced chemosensitivity to oxaliplatin. Unbiased transcriptomic analyses further corroborated that biological processes corresponding to “Apoptosis” were significantly downshifted in dormant µCRLM cultures and were restored upon escape and recovery from dormancy (Figure 5G).

## 3 Discussion

Clinical management of dormant colorectal cancer liver metastasis (CRLM) has been slowed by our limited understanding of these microscopic lesions. Due to their microscopic size, they are undetectable via standard diagnostic imaging, leaving little clinical insight to leverage for in vitro modeling. Consequently, our understanding of mechanisms that lead from dormancy to recurrent, chemoresistant metastases is severely limited. The impact of this limitation falls on patients who suffer with CRLM, especially males who make up the majority of colorectal cancer deaths despite similar incidence of CRLM in males and females.^[^ ^63^, ^64^, ^65^ ^]^ Future work should consider sex as a variable to study sex‐based determinants of dormancy behavior and reactivation.

Dormancy research in colorectal cancer has drawn extensively from work in other cancers like breast,^[^ ^20^, ^61^, ^66^, ^67^, ^68^, ^69^, ^70^, ^71^ ^]^ lung,^[^ ^72^, ^73^, ^74^ ^]^ and prostate^[^ ^21^, ^25^, ^26^, ^33^, ^75^ ^]^ to uncover mechanisms of induced dormancy in vitro. The complex dormancy phenotype is difficult to recapitulate in its entirety, though aspects of it have been successfully achieved, primarily through nutrient depletion^[^ ^40^, ^41^, ^42^ ^]^ and anticancer/chemotherapy drug treatment,^[^ ^43^, ^44^, ^45^, ^46^, ^47^, ^48^ ^]^ especially in 2D monolayer culture models. In our current work, we adopted both methods as a starting point to induce proliferative cell cycle arrest in 3D colorectal cancer spheroids (Figure 1), translating to reduced xenografted tumor growth rates in vivo (Figure 2). The growth rate is specifically mentioned since dormant tumors, initiated with fewer cells than the control group, not only exhibited delayed presentation but also showed persistently decreased growth rates after they appeared.

An emerging body of evidence suggests that dormancy induction and reversal can be orchestrated by the extracellular matrix (ECM) and its related biophysical cues,^[^ ^76^ ^]^ cell‐matrix adhesion interfaces,^[^ ^45^ ^]^ composition, and architecture.^[^ ^14^ ^]^ Monolayer cultures and even spheroid cultures do not replicate ECM‐based 3D cues, leaving a missed opportunity to study an important driver of dormancy in CRLM. Specific to CRLM, the liver ECM is a significant contributor to metastatic progression and chemoresistance.^[^ ^54^ ^]^ Given the central and vital role of the ECM, our current work aimed at engineering a dormant 3D in vitro ECM‐based model of colorectal cancer liver metastasis.

To provide the architecture, composition, and structural cues of the liver ECM, we leveraged intact decellularized ECM (dECM) scaffolds. These scaffolds were previously used to demonstrate that metastatic colorectal cancer spheroids seeded into dECM liver colonized scaffolds in an MMP9‐dependent manner.^[^ ^58^ ^]^ In our current study, we again aimed to engineer CRLM, but in its microscopic and dormant form. Our first step was to translate colorectal cancer spheroid dormancy, induced by nutrient depletion and low dose oxaliplatin chemotherapy, into the liver dECM scaffolds. Reducing serum levels in vitro emulates the stressful conditions experienced by disseminated cell clusters isolated in the liver without the vascular networks in place to support growth.^[^ ^77^, ^78^ ^]^ Low dose oxaliplatin chemotherapy can model clinical dormancy, as sublethal chemotherapy exposures foster resistant, dormant cell populations seen in minimal residual disease.^[^ ^79^, ^80^, ^81^, ^82^ ^]^ The 1 µm oxaliplatin used in our study is well below our spheroid IC_50_ (≈70 µM; Figure 5B) and aligns with concentrations commonly adopted in dormancy and resistance models.^[^ ^44^, ^83^ ^]^ Notably, clinical dosing ranges from 85‐130 mg m^−2^, which translates to single digit micromolar concentrations in patient plasma.^[^ ^84^, ^85^ ^]^ Our approach therefore reproduces drug exposures that cancer cells experience both in patients and established in vitro models. Furthermore, by modeling dormant, microscopic CRLM (µCRLM) with 10‐cell spheroids, we mimicked the nest sizes typical of clinical microscopic CRLM.^[^ ^86^, ^87^, ^88^, ^89^ ^]^

In line with dormancy, our data demonstrated that dormant spheroids colonized the dECM liver scaffolds but exhibited severely inhibited proliferation and growth rates (Figure 3A–D). In vivo tumor formation was observed for both control and dormant spheroids. Notably, dormant spheroids displayed a delayed tumor initiation compared to controls (Figure 2B); a phenomenon partially attributable to the lower initial cell count in dormant spheroid inoculations. However, even after accounting for this discrepancy in initial cell number, dormant tumors demonstrated markedly reduced growth rates post‐initiation compared to controls, highlighting a robust dormancy effect. We additionally assessed dormancy by targeted gene expression analysis of key cell cycle regulators, as well as unbiased transcriptomics. Our results demonstrated that while nutrient depletion and low dose oxaliplatin strongly induced G1/S cell cycle arrest (higher *TP53* (p53),^[^ ^90^, ^91^, ^92^, ^93^ ^]^ *CDKN1A* (p21)^[^ ^90^, ^91^, ^93^ ^]^ and *CDKN2B* (p15)^[^ ^92^, ^94^ ^]^ in Figure 1E), the introduction of the liver dECM variable otherwise induced and amplified G2/M‐based cell cycle arrest (decreased *CDC20, CDK1, AURKA;* Figure 3F; Figure S2, Supporting Information). This is unsurprising since in other cancer contexts, nutrient depletion and lung ECM aid in cell cycle arrest.^[^ ^72^, ^77^, ^95^ ^]^ The observed increase in *TP53* (p53) expectedly increased expression of its downstream target cyclin‐dependent kinase (CDK) inhibitor *CDKN2A* (p21). p15 can contribute to this signature by inhibiting cyclin D1‐CDK complexes necessary to progress past the G1 phase, suggesting the cyclin D1‐encoding gene, *CCND1*, whose transcript levels did not change in this model, is instead regulated at the protein level by upregulated p15 to contribute to dormancy.^[^ ^92^ ^]^ G2/M cell cycle arrest typically occurs when a cell responds to DNA damage repair.^[^ ^96^ ^]^ We believe that low dose oxaliplatin treatment stimulates the DNA damage response, which is amplified in colorectal cancer cells within the dECM liver scaffold, placing them in a prolonged state of G2/M arrest and dormancy, additionally evidenced by the transcriptomic signatures shown in Figure 4. µCRLM cultured for extended time points (day 15) within the dECM scaffold demonstrates upregulation of senescence associated secretory phenotype (SASP),^[^ ^97^, ^98^ ^]^ and DNA damage‐induced senescence.^[^ ^99^ ^]^ These changes are supported by previous work which characterized shifts in SASP over time, indicating the initial stress response of early senescence contrasted with later‐stage SASP upregulated for inflammatory and interferon signaling pathways needed for immune surveillance.^[^ ^100^ ^]^ The absence of strong changes in metabolic signatures is also unsurprising, as dormant cells are non‐proliferative and are thought to have active, but maladapted, metabolism to maintain survival.^[^ ^16^, ^101^ ^]^ This prolonged dormancy state was further demonstrated by its persistence through implantation and subsequent slowed growth in vivo (Figure S4, Supporting Information).^[^ ^102^ ^]^ dECM scaffold growth and transcriptomic data presented in Figures 3D–G and 4 indicated aspects of dormancy (proliferation, cell cycle dysregulation) altered as early as day 3 of scaffold culture. These data determined the in vivo experimental timeline, which involved mouse tumors generated with cells at that day 3 timepoint. Injection of mice with the day 15 late or deep senescence cells could interestingly modify the cellular growth patterns in vivo.

Next, we explored two key hallmarks of dormancy: i) reversibility;^[^ ^103^, ^104^ ^]^ and ii) resistance to chemotherapy. Restoration of nutrients and withdrawal of low dose oxaliplatin released the brakes on G1/S cell cycle checkpoints (Figure 3M) following with the data showing G1/S perturbation by these supplemental stimuli in spheroids (Figure 1E). However, the G2/M arrest signature found to be induced by the liver dECM itself (Figure 3F) remained even in recovered µCRLM (Figure 3N). This suggests that the liver dECM independently induces and/or maintains dormancy in CRLM. Reversal and recovery, however, were fully supported within the dECM scaffold, with an overt outgrowth of colorectal cancer cells following nutrient restoration (Figure 3J–L). This metric of reversibility was notable, since recurrent CRLM and overt metastases occur from microscopic dormant CRLM clinically. Though our in vivo experiments were not exogenously manipulated to evaluate dormancy reversal, some insights can be drawn from there, including the rationale for nutrient restoration to trigger escape and recovery. In vivo, dormant cells demonstrated decreased tumorigenicity in that their tumor presentation was delayed. When tumors grew, dormant tumors grew at slower rates than control tumors. Eventually, however, most tumors did grow and reach the tumor burden window following a period of dormancy (Figure 3I). Based on these data, it is likely that the cells achieved escape from dormancy to regain their proliferative activity. Literature suggests that escape from prolonged periods of quiescence in xenografted tumors is at least in part accompanied by increased angiogenesis and nutrient availability.^[^ ^105^, ^106^ ^]^ Longitudinal analyses of our in vivo model will be performed in future work to consider a molecular basis for this observed behavior to further develop an in vivo model of colorectal cancer dormancy.

Once CRLM recurs, it is not only aggressive but also significantly resistant to chemotherapy. Therefore, we evaluated the sensitivity of engineered dormant µCRLM to oxaliplatin. We first established an oxaliplatin IC_50_ in control and dormant spheroids, to determine drug dosing. As expected, dormant spheroids were more resistant to oxaliplatin treatment (Figure 5B). Enhanced resistance to chemotherapy is documented by several groups^[^ ^48^, ^107^, ^108^ ^]^ in disseminated and dormant colorectal cancer cells.^[^ ^109^ ^]^ Even in our own previous work on macroscopic proliferative metastases, larger colorectal cancer spheroids (100 cells/drop) were less resistant than the microscopic cell clusters evaluated here.^[^ ^58^ ^]^ Importantly, cytotoxic doses of oxaliplatin were administered to dormant cells 48 h following the previous low dose (1 µM) treatment. In vitro cultures have shown low drug availability and activity after 48 h even at high doses, indicating the majority clearance of the low dose drug prior to cytotoxic experiments in this case.^[^ ^110^ ^]^ In the dECM, a cytotoxic dose was determined empirically, starting at the ≈150 µM IC_50_ value of dormant spheroids (Figure 5B) to account for differences in diffusion across the 3D scaffold in accordance with literature demonstrating increased oxaliplatin tolerance in 3D ECM models.^[^ ^58^, ^85^ ^]^ The 300 µm dose was chosen to control but not completely overwhelm the µCRLM populations. Despite this increased dose of oxaliplatin, dormant µCRLM was significantly more resistant to oxaliplatin treatment compared to control non‐dormant CRLM (Figure 5D,E). These findings are in line with seminal literature that suggests that ECM inactivates pro‐apoptotic molecules, while inducing anti‐apoptotic molecules, to promote cell survival^[^ ^111^ ^]^ and resistance to chemotherapy.^[^ ^72^ ^]^ Indeed, our own unbiased transcriptomics demonstrated that biological processes associated with apoptosis are significantly downregulated following prolonged µCRLM culture and reversed upon removal of dormancy inducers (Figure 5G).

## 4 Conclusion

Microscopic dormant colorectal cancer liver metastases are a major obstacle driving disease recurrence and patient survival, as they remain clinically undetectable prior to delayed, aggressive outgrowth. Consequently, in vitro modeling is constrained by the absence of clinical insights into mechanisms or biomarkers of dormancy induction, maintenance, or reversal. Our liver dECM scaffold model, in combination with nutrient deprivation and low dose oxaliplatin chemotherapy, establishes a platform to study such mechanisms, importantly, in the context of the 3D liver ECM environment. The dormancy phenotype established in this work was characterized through metrics of cell cycle state, proliferation, tumorigenicity and chemoresistance. Even so, additional insights can be gained with improved visualization methods to study mechanisms promoting scaffold occupation, including tumor cell invasion, and matrix remodeling, to support and characterize a dormancy state. We recognize that conclusions from this model remain somewhat limited in their reach due to the presence of a simplified metastatic microenvironment, only consisting of ECM and cancer cells. However, this model was built to be intentionally modular to help decouple the intricacies of confounding tumor microenvironmental stimuli, but also to maintain the ability to add and scale complexity over time. As such, stromal and immune cells that contribute to clinical colorectal cancer dormancy, as well as precise control over aspects of matrix architecture and the presence of certain growth factors and other signaling molecules, will be future goals to include as layers of complexity within this model. Likewise, complementary in vivo models that recapitulate colorectal cancer metastases within the liver will be critical to validate, guide and refine in vitro approaches, shaping model design and experimental focus. As many mechanisms of dormancy and disease recurrence remain undiscovered, the advancement of biomimetic in vitro models will be paramount for discovering novel targets for detection and treatment in patients with dormant microscopic colorectal cancer metastases to prevent disease recurrence.

## 5 Experimental Section

### Materials

All biochemical and molecular biology reagents were purchased from ThermoFisher Scientific (RRID:SCR_008452) unless otherwise specified. Cell lines were purchased from the American Type Culture Collection (ATCC; RRID:SCR_001672). Ammonium hydroxide was obtained from RICCA Chemical Company. The platinum‐based chemotherapy drug oxaliplatin was purchased from MedChemExpress. The SYLGARD^TM^ 184 Silicone Elastomer Kit (DOW Chemical) was used for polydimethylsiloxane (PDMS) synthesis.

### 2D and 3D Cell Culture

The HCT116 adult male colon cancer cell line (RRID:CVCL_0291) was maintained in monolayer culture in Dulbecco's Modified Eagle Medium (DMEM) supplemented with 10% heat‐inactivated fetal bovine serum (FBS, Peak Serum, Inc.) and 1X Antibiotic‐Antimycotic solution at 37 °C with 5% CO_2_. HCT116 colorectal cancer spheroids were formed on a 384‐well hanging drop array based on well‐established protocols.^[^ ^58^, ^62^, ^112^, ^113^, ^114^, ^115^ ^]^ 10 HCT116 cells were seeded in a 20 µL drop of growth medium and maintained for up to 9 days, with visual confirmation of spheroid formation by phase contrast microscopy.

### Spheroid Dormancy and Growth

Dormancy was induced in HCT116 cells by initiating spheroids with low (2%) serum medium and maintaining the cells in this nutrient‐depleted state over the course of 6 days. Control samples were maintained in complete (10% serum) medium for the duration of the experiments. On day 4 of spheroid culture, 1 µM oxaliplatin prepared in the corresponding serum‐containing medium (2% or 10%), was administered to the spheroids. To monitor growth, spheroids were collected on days 0, 1, 4, and 6 and mechanically dissociated. Cell counts were performed using a hemocytometer and normalized to the number of spheroids collected. Spheroid size was also analyzed in phase contrast micrographs. The 2D area projection of the spheroids was outlined by hand in the NIH ImageJ Software (RRID:SCR_003070) to measure spheroid area across multiple timepoints.

### Flow Cytometry

HCT116 spheroids were harvested for flow cytometry on day 6 of spheroid culture. Following mechanical dissociation, single‐cell suspensions were incubated with anti‐Ki67 antibodies tagged with AlexaFluor 647 (BD Biosciences; RRID:AB_647130) for 30 min at 37 °C. Matched isotype controls were used to establish background. Flow cytometry data were acquired using an Attune NxT Flow Cytometer (ThermoFisher Scientific; RRID:SCR_019590) and analyzed using FlowJo (RRID:SCR_008520) with a background cutoff at 0.5%.

### RNA Extraction and qPCR

RNA was extracted using the RNeasy Micro Kit (Qiagen; RRID:SCR_008539). Tissue samples (see sections 5.6 for scaffolds and 5.11 for tumors) were first dissociated by mincing in TRIzol^TM^ LS Reagent and then processed with the same kit following manufacturer's protocols. RNA concentration and purity were measured with a NanoDrop OneC instrument (ThermoFisher Scientific; RRID:SCR_023005). Reverse transcription was performed using the Applied Biosystems High Capacity cDNA Reverse Transcription Kit. cDNA was amplified for the genes of interest using the primers listed in **Table** **1** and PowerUp SYBR Green PCR Master Mix for detection on a QuantStudio 3 Real‐Time PCR System (Applied Biosystems; RRID:SCR_019712). *GAPDH* was used as a housekeeping gene to allow for analysis with the 2ΔΔC_t_ method.

**Table 1:** Primer sequences for genes analyzed with qPCR.

Gene	Forward primer sequence	Reverse primer sequence
***GAPDH***	CTGGGCTACACTGAGCACC	AAGTGGTCGTTGAGGGCAATG
***TP53***	CAGCACATGACGGAGGTTGT	TCATCCAAATACTCCACACGC
***CDKN1A***	TGTCCGTCAGAACCCATGC	AAAGTCGAAGTTCCATCGCTC
***CDKN2B***	ACGGAGTCAACCGTTTCGGGAG	GGTCGGGTGAGAGTGGCAGG
***CCND1***	GCTGCGAAGTGGAAACCATC	CCTCCTTCTGCACACATTTGAA
***CDC20***	GCACAGTTCGCGTTCGAGA	CTGGATTTGCCAGGAGTTCGG
***CDK1***	AAACTACAGGTCAAGTGGTAGCC	TCCTGCATAAGCACATCCTGA
***AURKA***	GAGGTCCAAAACGTGTTCTCG	ACAGGATGAGGTACACTGGTTG
***SLC2A1***	GGCCAAGAGTGTGCTAAAGAA	ACAGCGTTGATGCCAGACAG
***PDK1***	GGATTGCCCATATCACGTCTTT	TCCCGTAACCCTCTAGGGAATA
***G6PD***	ACCGCATCGACCACTACCT	TGGGGCCGAAGATCCTGTT

### Microscopic Colorectal Cancer Liver Metastasis (µCRLM) Generation

A µCRLM model was generated by adapting the previously published approach, which combines tumor spheroids with decellularized tissue as a metastasis model.^[^ ^58^ ^]^ Porcine livers were acquired from Rosenthal Meat Center in excess. The whole livers were sectioned and decellularized using a series of water and TritonX‐100/ammonium hydroxide detergent washes along with DNAse enzymatic treatment. Decellularized extracellular matrix (dECM) sections were lyophilized to generate shelf‐stable scaffolds with intact original liver architecture. 0.5–1.0 mg dECM scaffolds were rehydrated in serum‐free medium and secured with a pin to the center of each well of PDMS‐coated well‐plates. On day 6 of spheroid growth, 10 HCT116 spheroids were collected in a tube, mechanically dissociated to form smaller cell clusters, and seeded onto each liver scaffold. The cells were incubated for 2 h to allow for adherence to the scaffold and then submerged in medium for continued culture. Spheroids initiated with complete medium served as control samples which were maintained in complete medium throughout dECM culture, as well. Spheroids which were made dormant in hanging drop culture (see section 5.3) were contrarily submerged in dormancy medium (2% FBS + 1 µM oxaliplatin) for the duration of the µCRLM experiments. Medium on all samples was changed every 3‐4 days and samples were collected on days 0, 3, 10 and 15 for downstream analysis including overnight fixation with 4% paraformaldehyde (Santa Cruz Biotechnology; RRID:SCR_008987) for microscopy.

### µCRLM Imaging—Scanning Electron Microscopy (SEM)

Fixed samples were dehydrated with ethanol and lyophilized for 48 h. Gold coating was performed with a Cressington Vacuum Sputter Coater 108 (RRID:SCR_019824) to prepare samples for imaging with a Quanta 600 Field Emission‐Scanning Electron Microscope (FEI Company). Cells in SEM images were pseudocolored maroon using Adobe Photoshop.

### Light Sheet Fluorescence Microscopy

Following fixation, µCRLM samples were processed and imaged according to protocols previously optimized for dECM scaffold visualization.^[^ ^57^ ^]^ Briefly, tissue clearing was performed by passing samples through a dehydration series, followed by incubation in dichloromethane and refractive index matching with ethyl cinnamate. Once cleared, cells were labeled with a BioTracker Red fluorescent membrane dye (Sigma Aldrich, RRID:SCR_008988) and imaged using the ZEISS Z.7 light sheet microscope (RRID:SCR_024448). Raw image data was processed with an optimized analysis protocol^[^ ^57^ ^]^ using ImageJ, Imaris (RRID:SCR_007370) and MATLAB (RRID:SCR_001622) software. Representative 3D volume images were pseudocolored and captured in Imaris.

### µCRLM Growth

The CellTiter Glo Luminescent Viability ATP reporter assay (Promega Corporation; RRID:SCR_006724) was performed to measure viability over time of paired samples at days 0, 3, 10 and 15 of µCRLM culture. At the timepoint of interest, each scaffold was transferred into a 1:1 solution of CellTiter Glo reagent and culture medium in an opaque‐walled 96‐well plate. Per the manufacturer's protocols, the samples and solutions were mixed by 2 min of shaking to lyse the cells followed by a 10‐min incubation and luminescence recording on a BioTek Cytation 7 microplate reader (RRID: SCR_019733). Each reported value was normalized to a parallel day 0 luminescence measurement for time‐dependent analysis of proliferation.

### RNA Sequencing

RNA was extracted from spheroids and µCRLM scaffolds as described in section 5.5. Total RNA integrity was determined using the Agilent 4200 Tapestation (RRID:SCR_018435). Library preparation was performed with 10 ng of total RNA with a Bioanalyzer RIN (RNA integrity number) score greater than 8.0. ds‐cDNA was prepared using the SMARTer Ultra Low RNA kit for Illumina Sequencing (Takara Bio, Inc., RRID:SCR_021372) per manufacturer's protocol. Raw data were sequenced on an Illlumina NovaSeq X Plus (RRID:SCR_024568) and aligned to the Ensembl release 101 primary assembly with STAR version 2.7.9a1. Gene counts were derived from the number of uniquely aligned unambiguous reads by Subread:featureCount version 2.0.32.

### Analysis and Visualization of Sequenced RNA Data

Raw count data from RNA sequencing experiments were preprocessed using R (v4.2.2) and associated Bioconductor packages (RRID:SCR_006442). Differential gene expression analysis was conducted using preprocessed count matrices from the various comparisons across conditions, including removal of metadata columns and transformation steps for downstream analysis. Gene‐level counts were filtered and used to calculate log2fold changes (logFC) and p‐values. Genes with a p‐value < 0.05 and absolute logFC ≥ 2 were considered significantly differentially expressed. Volcano plots were generated using ggplot2 (R package; RRID:SCR_014601).

Differentially expressed genes with a *p*‐value < 0.05 and logFC of +/‐ 2.0 were compared between day 3 and day 15 conditions and visualized as a Venn diagram. Results were used to identify unique genes for each condition both up‐ and down‐regulated. The list of unique genes was then evaluated using Enrichr (RRID:SCR_001575).^[^ ^116^, ^117^, ^118^ ^]^ Pathway enrichment was assessed against the Reactome 2024 gene set found in the Enrichr database and the top 10 enriched processes were identified. For the Recovery dataset, all differentially expressed genes with a p value < 0.05 and logFC of +/‐ 2.0 were loaded into Enrichr to determine the top 10 enriched pathways both up‐ and down‐regulated.

Pathway enrichment analysis was performed for direct comparison across all conditions using the Camera (Correlation Adjusted M Ean RAnk) method in the Reactome database (RRID:SCR_003485).^[^ ^119^ ^]^ Camera analysis was applied to raw count data and normalized using the Trimmed Mean of M‐values (TMM) method.^[^ ^120^ ^]^ Pathway enrichment was assessed while adjusting for correlation structures within gene sets, thereby reducing bias in enrichment scores. Statistical significance was determined using competitive gene set testing, and pathways with a false discovery rate (FDR) < 0.05 were considered significantly enriched. All analyses were conducted using the limma package in R, and Voronoi visualizations were generated using Reactome's online tools. Results from Reactome Camera analysis were also displayed as dot plot graphics for direct comparison of selected gene sets between experimental groups.

### Oxaliplatin Chemotherapy Treatment

Oxaliplatin was administered to hanging drop spheroids on day 6 of culture by dilution in each condition's respective culture medium (10% or 2% FBS). Each spheroid was fed with 2 µL of drug‐containing medium to dilute 1:10 in the 20 µL drop and reach final concentrations of 0‐500 µM. Following 48 h of incubation with the drug, spheroids were collected, and their viability was assessed with the CellTiter Glo Assay (see section 5.8). Normalized percent viability values were determined by comparing to untreated controls, and IC_50_ values calculated using the normalized log(Inhibitor) Variable Slope Nonlinear Fit function in GraphPad Prism.

µCRLM samples were seeded as described in section 5.6. To test µCRLM chemoresponse, oxaliplatin was administered in fresh medium (control or dormancy) at 300 µm on day 3. The samples were allowed to incubate with the drug for 48 h prior to collection for viability analysis. Treated samples were compared to untreated controls to determine percent viability.

### In Vivo µCRLM Establishment in NOD/SCID Gamma Mice

Male NOD.Cg‐*Prkdc^scid^Il2rg^1Wjl^*/SzJ (NOD/SCID gamma; RRID:BCBC_4142) mice ages 7‐8 weeks old were obtained from The Jackson Laboratory (RRID:SCR_004633). Mice were housed at Texas A&M University and all procedures conducted within approved Texas A&M University Institutional Animal Care and Use Committee guidelines (Protocol 2024‐0040 D). For spheroid injections, day 9 HCT116 spheroids were collected from hanging drop culture, mechanically dissociated and resuspended in phosphate buffered saline (PBS; Figure 2A). For µCRLM injections, recellularized scaffolds were collected on day 3 of µCRLM culture and minced in PBS (Figure 3H). Matrigel was added (1:1) to suspend either 10 spheroids or 1 µCRLM scaffold (0.5–0.6 mg) in 100 µL of volume. Tumors were established by injecting either spheroids or minced µCRLM scaffolds subcutaneously into both flanks of the mice using a 21G needle to deliver 100 µL per flank. Mouse weight was monitored three times weekly and injection sites were palpated for tumor presence. Once palpable, tumor dimensions were measured with calipers and volume calculated with Equation (1):

(1) $$Volume=\frac{\left(majoraxis\times minoraxis^{2}\right)}{2}$$

Mice were euthanized upon reaching a 2000 mm^3^ total tumor burden per mouse (sum of volumes of two subcutaneously injected flank tumors). Tumors were excised and processed for RNA extraction and qPCR analysis (see section 5.5).

### Statistics

GraphPad Prism 10 software (RRID:SCR_002798) was used to perform statistical analysis. Resulting values from these analyses were reported as the mean and standard error of the mean from 3‐5 biological replicates per condition for each experiment. Mouse experiments were conducted with 3–6 mice per group, with 2 flank tumors in each mouse. Oxaliplatin IC_50_ values for spheroids were calculated by normalizing luminescence values from the CellTiter Glo assay to untreated controls. Similarly, fold changes in proliferation within µCRLM samples were generated by normalizing luminescence values to a paired day 0 control for each condition. Drug response in µCRLM was reported as percent viability, as luminescence values from oxaliplatin treated samples were normalized to untreated controls within each condition (control or dormant) at the same timepoint. All qPCR data was also compared to control conditions within the same experiment and performed in triplicate over at least three biological replicates. One‐way ANOVA and two‐way ANOVA analyses were performed as appropriate. Statistical significance was indicated with associated p‐values for each data set, where a p‐value less than 0.05 was considered significant.

## Conflict of Interest

The authors declare no conflict of interest.

## Author Contributions

S.N.V. performed conceptualization, data curation (lead), formal analysis (lead), investigation (lead), methodology (lead), visualization (lead), wrote‐original draft, wrote‐review, and edited. L.L.N. performed investigation (supporting), methodology (supporting), visualization (supporting); A.J.C. performed formal analysis (supporting), visualization (supporting). C.A.C. performed data curation (supporting), visualization (supporting). O.R.B. performed investigation (supporting), software. S.R. performed investigation (supporting). B.G., A.S., and A.T. performed investigation (supporting). S.H. performed visualization (supporting). A.N.S. managed resources. S.K. performed conceptualization (co‐lead), wrote‐review, and edited (supporting). A.J.W. performed investigation (supporting), funding acquisition (supporting), resources (co‐lead), wrote‐review, and edited (supporting). S.A.R. performed conceptualization (lead), funding acquisition (lead), formal analysis (supporting), methodology (supporting), project administration (lead), supervision (lead), writing‐review and editing (lead).

## Supporting information



Supporting Information

## Data Availability

The data that support the findings of this study are openly available in the Raghavan Lab Dataverse within the Texas Data Repository at https://doi.org/10.18738/T8/ZGSYEP.
