# Supplementary material for: Decellularized Extracellular Matrix Scaffolds to Engineer the Dormant Landscape of Microscopic Colorectal Cancer Liver Metastasis
Source: Adv Healthc Mater. 2025 Oct 13;15(8):e01791. doi: 10.1002/adhm.202501791 (PMC12927541; doi:10.1002/adhm.202501791)
Supplement: Supplementary file 1 — Supporting Information [file ADHM-15-0-s001.pdf]

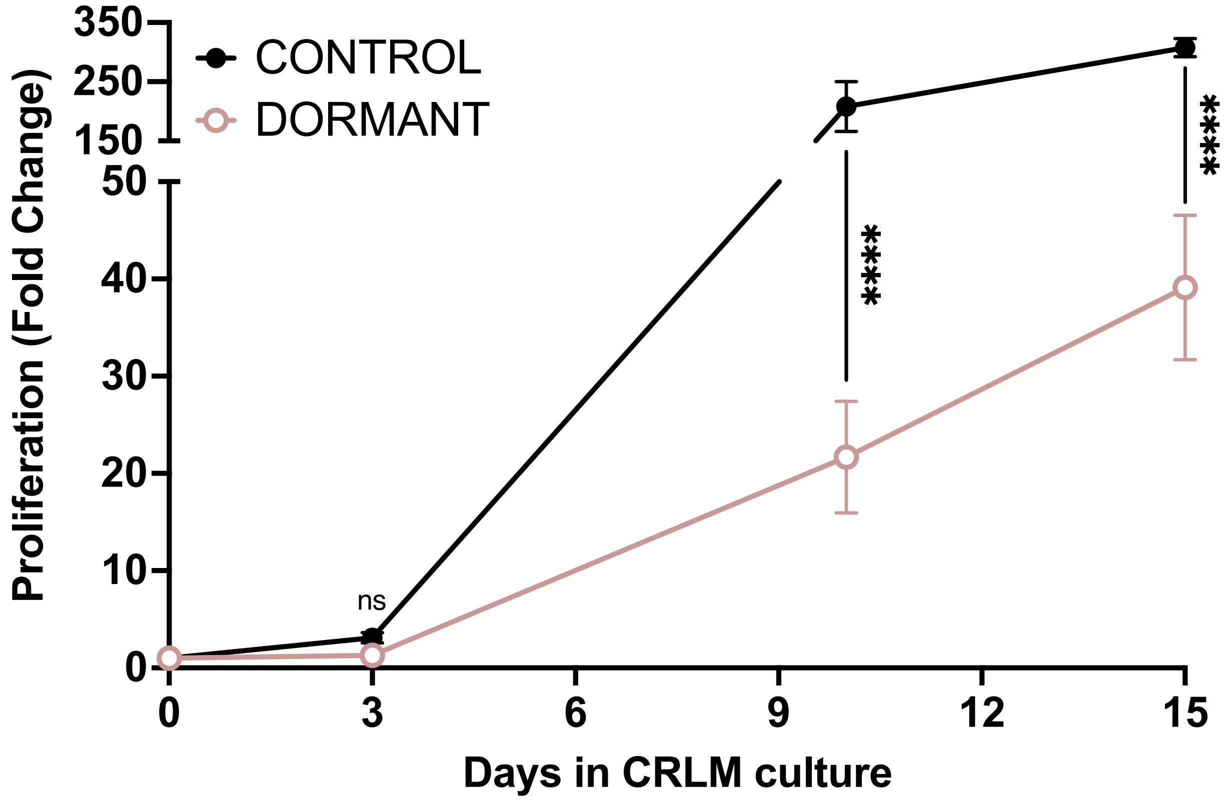

Figure S1. Proliferation within µCRLM scaffolds seeded with equal cell numbers. Control and dormant µCRLM were initiated with 569 cells, corresponding to the average number of cells for dormant spheroid recellularization (Figure 1C). Proliferation was assessed over time with the CellTiter Glo assay. RLU values were background-subtracted and reported as fold change relative to day 0. Despite equal initial seeding, dormant scaffolds still exhibited reduced proliferation as early as day 3 (1.31 ± 0.18-fold vs. 3.11 ± 0.53-fold in controls; ns, two-way ANOVA) with significance reached by day 10 when control µCRLM reached 208.17 ± 42.23-fold and dormant scaffolds demonstrated only 21.67 ± 5.75-fold (****$p<0.0001$, two-way ANOVA).

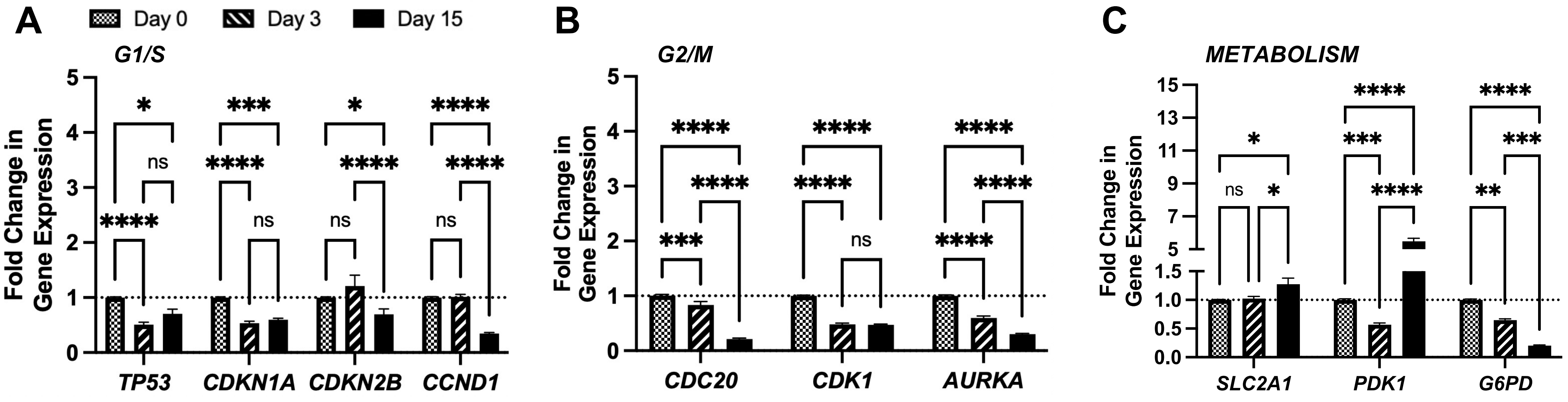

Figure S2. dECM scaffold influence on control cells. μCRLM generated with control spheroids (day 0) were assessed for cell cycle arrest signatures using qPCR. A) G1/S phase regulatory gene expression was mildly impacted by the introduction of dECM, with the most prominent change being a 0.35 ± 0.02-fold decrease in *CCND1* at day 15 (****p<0.0001, two-way ANOVA). B) G2/M arrest signatures were induced in response to dECM through a decrease in expression of *CDC20*, *CDK1*, and *AURKA* over time, with day 15 showing the strongest arrest signatures (****p<0.0001, two-way ANOVA). C) Metabolic gene expression was also impacted in μCRLM, exhibiting a 5.49 ± 0.18-fold increase in *PDK1* (****p<0.0001, two-way ANOVA) and a 0.20 ± 0.01-fold decrease in *G6PD* (****p<0.0001, two-way ANOVA), suggesting that the cells may experience dysregulated metabolism in response to dECM.

		DAY 3		DAY 15 *Dormant*		DAY 15 *Recovery*	
DOWN / UP							
**Gene**	**Reactome Pathways**	**LogFC**	**p-value**	**LogFC**	**p-value**	**LogFC**	**p-value**
TP53	*Cellular Senescence*<br>*Cell Cycle*<br>*Metabolism of Proteins*	**- 1.121**	0.0329	**1.060**	0.0290	**-0.899**	0.0549
CDKN1A	*Cellular Senescence*<br>*Cell Cycle*<br>*Metabolism of Proteins*	**0.616**	0.3280	**1.690**	0.0083	**- 5.190**	0.0459
CDKN2B	*Cellular Senescence*<br>*Cell Cycle*	**0.538**	0.2850	**0.943**	2.5E-06	**- 1.130**	6.5E-07
CCND1	*Cell Cycle*	**0.413**	7.2E-08	**- 0.365**	8.8E-07	**- 0.104**	0.0367
AURKA	*Cell Cycle*<br>*Metabolism of Proteins*	**0.721**	0.0031	**- 0.852**	0.0322	**-**	-
CDC20	*Cell Cycle*<br>*Metabolism of Proteins*	**- 0.280**	0.4359	**-**	-	**- 0.645**	0.0861
PDK1	*Metabolism of Proteins*	**1.159**	0.0119	**0.955**	5.0E-05	**1.120**	0.0122

Figure S3. Dormancy genes of interest observed through RNAseq analysis. Genes of interest from targeted qPCR gene expression analysis (Figures 1-3) were also identified in the Reactome analysis of bulk RNA sequencing data. Genes associated with G1/S cell cycle phase (*TP53*, *CDKN1A*, *CDKN2B*, *CCND1*) and G2/M phase (*AURKA*, *CDC20*) arrest were associated with the “Cellular Senescence” and “Cell Cycle” pathways. *AURKA*, *CDC20,* and *PDK1* were also found within “Metabolism of Proteins”. LogFC and p-values are reported for each gene as compared to the day 0 (dormant spheroids, scaffold-naïve) condition. Overall expression trends in these genes mirrored those measured by qPCR for days and 3 and 15 of µCRLM. We observed mixed expression of *TP53*, strong *CDKN2B* upregulation, reduced *CDC20*, and increased *PDK1*. Further, following recovery from dormancy (right column), all the cell cycle arrest genes were downregulated, and *PDK1* was increased, matching trends shown in the qPCR data of Figure 3.

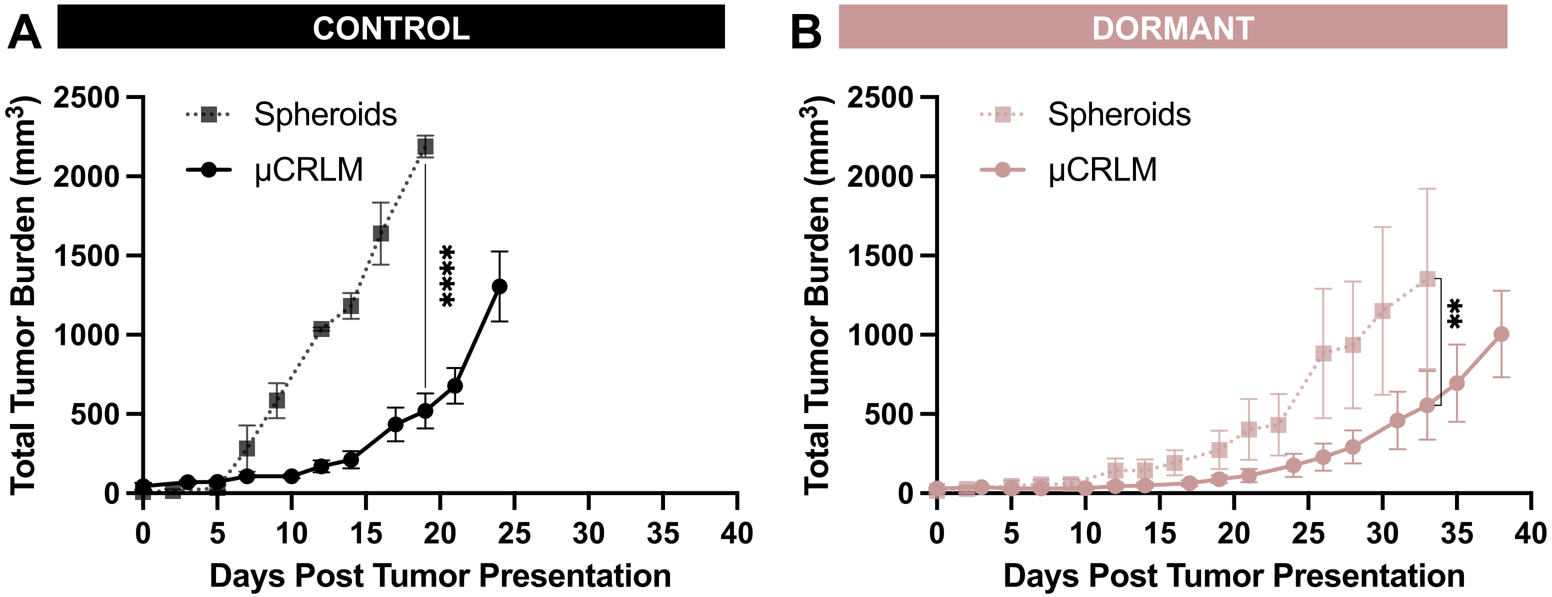

Figure S4. In vivo functional dormancy induced by dECM scaffolds. Tumors were initiated in mice by injecting either 10 spheroids or 1 μCRLM scaffold into each flank to establish two tumors per mouse. The total tumor burden reports the sum of the tumor volumes of both tumors on the same mouse, for 3-6 mice per group. Tumor growth curves were aligned to the first day of tumor presentation within each group (designated as day 0). For each group, data were plotted until the day that group reached endpoint criteria and statistical significance is displayed for the corresponding timepoint. A) Tumors established from control scaffolds grew significantly slower than spheroid-cultured tumor cells (day 19; ****$p<0.0001$, two-way ANOVA). B) In light of the already dampened growth of dormant cells in vivo, a smaller difference was observed between spheroid-generated tumors as compared to those established from μCRLM scaffolds. Still, tumor growth was significantly dampened in the μCRLM condition (day 33; **$p<0.005$, two-way ANOVA).
